# Evolution and spread of glyphosate resistance in *Conyza canadensis* in California

**DOI:** 10.1111/eva.12061

**Published:** 2013-03-11

**Authors:** Miki Okada, Bradley D. Hanson, Kurt J. Hembree, Yanhui Peng, Anil Shrestha, Charles Neal Stewart, Steven D. Wright, Marie Jasieniuk

**Affiliations:** ^1^ Department of Plant Sciences Mail Stop 4 University of California, Davis Davis CA USA; ^2^ University of California Cooperative Extension Fresno County Fresno CA USA; ^3^ Department of Plant Sciences University of Tennessee Knoxville TN USA; ^4^ Department of Plant Science California State University, Fresno Fresno CA USA; ^5^ University of California Cooperative Extension Tulare and Kings Counties Tulare CA USA

**Keywords:** agricultural weed, *Conyza canadensis*, *Erigeron canadensis*, glyphosate, herbicide resistance, microsatellite marker

## Abstract

Recent increases in glyphosate use in perennial crops of California, USA, are hypothesized to have led to an increase in selection and evolution of resistance to the herbicide in *Conyza canadensis* populations. To gain insight into the evolutionary origins and spread of resistance and to inform glyphosate resistance management strategies, we investigated the geographical distribution of glyphosate resistance in *C. canadensis* across and surrounding the Central Valley, its spatial relationship to groundwater protection areas (GWPA), and the genetic diversity and population structure and history using microsatellite markers. Frequencies of resistant individuals in 42 sampled populations were positively correlated with the size of GWPA within counties. Analyses of population genetic structure also supported spread of resistance in these areas. Bayesian clustering and approximate Bayesian computation (ABC) analyses revealed multiple independent origins of resistance within the Central Valley. Based on parameter estimation in the ABC analyses, resistant genotypes underwent expansion after glyphosate use began in agriculture, but many years before it was detected. Thus, diversity in weed control practices prior to herbicide regulation in GWPA probably kept resistance frequencies low. Regionally coordinated efforts to reduce seed dispersal and selection pressure are needed to manage glyphosate resistance in *C. canadensis*.

## Introduction

Agricultural weeds regularly adapt to multiple selective pressures at the contemporary timescale, including climate and soil conditions (e.g., Begg et al. 2012) as well as crop, soil, and weed management practices (e.g., Barrett 1983; Powles and Yu 2010; Owen et al. 2011). The capacity of weeds for rapid adaptation poses constant challenges for farmers and land managers because weeds reduce production in agriculture and forestry and are expensive to manage with damages estimated to total $37 billion annually in the USA (Pimentel et al. 2000, 2005). One of the best examples of weed adaptation to management practices is the evolution of resistance to herbicides (Maxwell et al. 1990; Jasieniuk et al. 1996; Powles and Yu 2010). Herbicide resistance is defined as ‘the inherited ability of a plant to survive and reproduce following exposure to a dose of herbicide normally lethal to the wild type’ (WSSA 1998). Resistance has evolved multiple times in weed species and to many herbicides, including glyphosate. To date, weed populations with glyphosate‐resistant individuals have been identified in 24 species worldwide (Heap 2012). Glyphosate has several favorable properties as a herbicide, including low mammalian toxicity, very low activity in the soil, and effectiveness on a diversity of species, which have made it a key weed management tool in modern agriculture (Baylis 2000; Duke and Powles 2008). Glyphosate use in agriculture has increased markedly in the last two decades due to the adoption of no‐tillage and reduced tillage practices as well as the introduction of transgenic glyphosate‐resistant crops (Owen 2008; Powles 2008). Continued evolution of weed resistance to glyphosate threatens sustained use of this economically important herbicide.

In *Conyza canadensis*, resistance to glyphosate is widespread with populations confirmed to contain resistant individuals in 20 states of the USA and five countries worldwide (Heap 2012). Resistant individuals of *C. canadensis* were first identified in a transgenic glyphosate‐resistant soybean field in the state of Delaware, USA, following only 3 years of repeated glyphosate use (VanGessel 2001). In California, glyphosate‐resistant individuals were first confirmed in 2005 in the Central Valley (Shrestha et al. 2007) where glyphosate has been the primary herbicide used for weed control in orchards, vineyards, field edges, roadsides, and irrigation ditches for decades (CADPR 2009). Recent entry of lower cost, generic glyphosate into the market has undoubtedly increased the reliance on glyphosate in California. However, one of the main factors hypothesized to underlie the evolution of glyphosate‐resistant *C. canadensis* in California is the increased use of glyphosate following the implementation of state regulations restricting certain pesticides vulnerable to leaching and runoff into groundwater (CADPR 2004; Shrestha et al. 2007). To design weed management strategies that prevent the continued evolution and spread of glyphosate‐resistant weeds, information on the factors that may have increased selection for resistance and the evolutionary and demographic histories of glyphosate‐resistant populations, including their origins and geographical pathways of spread, is critical. Such information will indicate whether glyphosate resistance originated once and spread from a single source population or originated multiple independent times within distinct populations, and whether increased selection for resistance may have occurred. If resistance originated once and spread, reduction or prevention of seed dispersal from resistant populations will be required to prevent or slow resistance evolution. Alternatively, if resistance originated multiple times and spread from multiple sources, reduction in both seed dispersal and selection pressure will be needed. If glyphosate resistance evolved only once and spread widely, resistant individuals may be contained eventually with both tactics. However, if resistance is likely present within any given population and can evolve multiple times independently within a region, glyphosate should always be used as a part of integrated weed management approaches within a region to prevent independent origins.

During the evolution and spread of weed resistance to herbicides, multiple mutations conferring resistance, strong positive selection, population bottlenecks, and founder events not only determine the spatial structuring of phenotypic variation across an agricultural landscape (Jasieniuk et al. 1996; Neve et al. 2009), but also shape neutral genetic variation within and among populations (Charlesworth et al. 2003). Together, spatial patterns of adaptive phenotypic variation (resistance and susceptibility of individuals to herbicide) and population genetic structure provide information on the sources of resistant plants, pathways and demographic processes underlying resistance spread, and the environments strongly selecting for resistance. Additionally, Bayesian coalescent‐based approaches, such as approximate Bayesian computation (ABC) analysis (Beaumont et al. 2002; Estoup and Guillemaud 2010), can provide further insight into the dynamics of resistance spread by indicating whether resistance evolved once and spread to other populations or evolved independently multiple times in multiple populations, based on the relative probabilities of explicitly stated competing scenarios. The ABC approach to inferring the origins of herbicide‐resistant populations is particularly useful when the molecular genetic basis of resistance is unknown and thus the DNA sequence variation in the gene that confers resistance cannot be investigated. ABC analysis also enables estimation of the timing of past changes in the effective sizes (*N*_*e*_) of weed populations, which may reflect the timing of selection and resulting reduction in *N*_*e*_, or the timing of resistance evolution and subsequent increase in *N*_*e*_. As the timing of glyphosate use in agriculture is known, estimation of the timing of changes in *N*_*e*_ using ABC analysis may allow insights into the impact of glyphosate on the *N*_*e*_ of weed populations and the timing of the evolution of resistance to glyphosate. Such insights into the evolutionary and demographic processes underlying the origins and spread of herbicide‐resistant weed populations in an agricultural landscape are essential for the design of weed resistance management strategies.

In this study, we examined the evolutionary origins and spread of glyphosate resistance in *C. canadensis* populations within orchards and vineyards across the Central Valley of California and in other human‐disturbed habitats surrounding the valley. We analyzed plant response to glyphosate and microsatellite marker variation in each population and addressed the following five questions: (i) how is glyphosate resistance distributed across the area sampled? (ii) is the distribution of glyphosate resistance correlated to the distribution of groundwater protection areas (GWPA)? (iii) is there spatial structuring of population genetic variation and multilocus genotypic variation associated with glyphosate resistance? (iv) are there distinct populations of glyphosate‐resistant plants that evolved resistance independently? (v) can we detect changes in *N*_*e*_ by ABC analysis that correspond to the timing of selection by glyphosate or the timing of evolution of glyphosate resistance? and (vi) how do the observed spatial patterns of phenotypic and genetic variation inform the design of strategies that slow or prevent the further evolution and spread of weed resistance to glyphosate?

## Materials and methods

### Study species

*Conyza canadensis* (L.) Cronquist (synonym: *Erigeron canadensis* L.; common names: horseweed, marestail) is native to North America (Noyes 2000) and occurs worldwide but most commonly in temperate areas (Weaver 2001). *Conyza canadensis* is an early successional winter and summer annual commonly found in orchards, vineyards, arable fields with reduced or no‐tillage, pastures, rangeland, roadsides, railroads, and canal banks (Weaver 2001). *Conyza canadensis* has a highly self‐fertilizing mating system and reproduces only by seed (Weaver 2001). Each plant is capable of producing over 200 000 wind‐ and/or water‐dispersed seeds (Davis et al. 2009), which regularly disperse within 100 m but up to 500 m from source populations (Dauer et al. 2007) and can travel 2–122 km (Dauer et al. 2009a). In *C. canadensis*, glyphosate resistance segregates as an incompletely dominant trait that is controlled by a single major locus (Zelaya et al. 2004) although the molecular genetic basis of the mechanism has not been identified.

### Sampling

**Figure 1:**
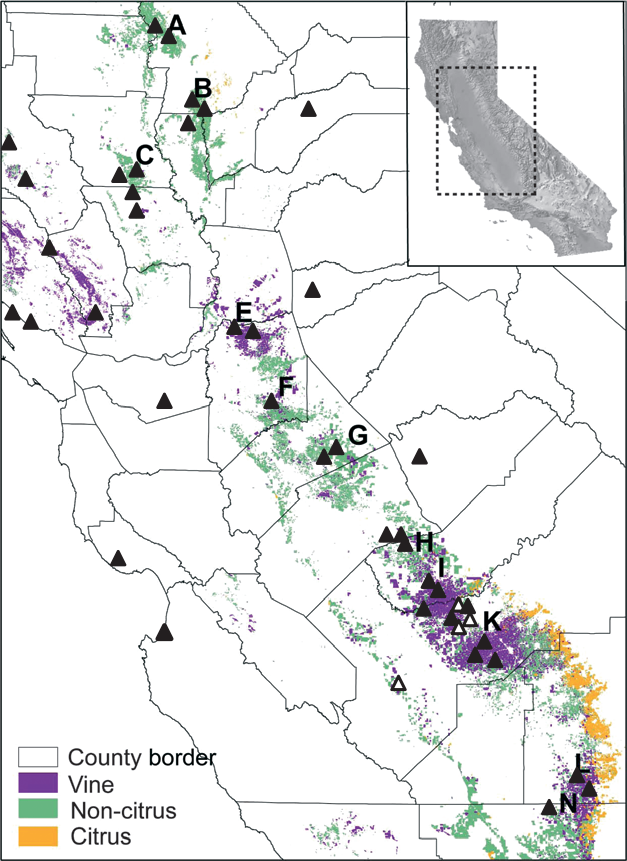
Geographical distribution of sampled populations of *Conyza canadensis* and perennial cropping systems. Closed triangles indicate populations sampled for leaf tissue and seed used in microsatellite marker genotyping and assessment of response to the glyphosate, respectively; open triangles indicate populations sampled only for leaf tissue.

Forty‐two populations of *C*. *canadensis* were sampled in 2010 across the Central Valley of California, including 30 populations from orchards and vineyards and 12 populations from other human‐disturbed habitats (Fig. 1, Table 1). Within each population, leaf tissue was collected from 30 plants selected haphazardly while walking parallel transects across the sampling area. Seeds were also collected from each plant in 38 of the 42 populations. Plants in the remaining four populations were prereproductive at the time of leaf sampling and thus were not sampled for seed. In addition to the plants sampled in the field, we collected leaf tissue from 30 plants each grown from seed collected from a glyphosate‐susceptible and a glyphosate‐resistant population previously characterized (Shrestha et al. 2007). Sampled leaf tissue was immediately dried in sealed plastic bags filled with silica gel and stored at room temperature until DNA extraction.

**Table 1:** *Conyza canadensis* populations and geographical regions sampled in California and the frequencies of plants that survived glyphosate treatment in each. Populations are sorted by latitude and habitat type

Pop'n ID	Population habitat	Latitude (N)	Longitude (W)	No. plants treated	No. plants surviving	*R*	SE	*R* per region	County	GWPA (km^2^)
Northern region										
A1	Orchard: prune	39.589	121.801	89	3	0.03	0.020	0.07	Butte	3
A2	Orchard: almond	39.714	121.843	60	4	0.07	0.067		Butte	3
B1	Orchard: walnut	39.353	121.726	60	8	0.13	0.133		Butte	3
B2	Orchard: walnut	39.331	121.678	52	0	0.00	0.000		Butte	3
B3	Orchard: almond/walnut	39.106	121.689	50	1	0.02	0.017		Sutter	224
C1	Orchard: almond	38.951	122.060	40	0	0.00	0.000		Colusa	5
C2	Orchard: almond	39.012	122.070	60	1	0.02	0.017		Colusa	5
YOL2	Orchard: peach	38.707	122.075	60	14	0.23	0.200		Yolo	78
SON1	Vineyard: grape	38.671	122.813	78	10	0.11	0.059		Sonoma	36
Central region										
E1	Vineyard: grape	38.169	121.202	50	27	0.53	0.075	0.68	San Joaquin	683
E2	Vineyard: grape	38.190	121.417	54	54	1.00	0.000		San Joaquin	683
F2	Orchard: cherry	37.821	121.108	51	50	0.98	0.024		San Joaquin	683
G2	Orchard: almond	37.610	120.755	67	59	0.86	0.055		Stanislaus	1219
G3	Orchard: almond	37.551	120.811	82	3	0.04	0.038		Stanislaus	1219
Southern region										
H1	Orchard: almond	37.040	120.221	51	39	0.78	0.087	0.88	Madera	523
H2	Orchard: pomegranate	36.996	120.241	56	55	0.95	0.048		Madera	523
H3	Vineyard: grape	37.014	120.230	89	88	0.99	0.011		Madera	523
I1	Vineyard: grape	36.824	120.187	42	42	1.00	0.000		Madera	523
I2	Vineyard: grape	36.938	120.129	71	41	0.53	0.097		Madera	523
I3	Vineyard: grape	36.982	120.202	60	51	0.85	0.150		Madera	523
K1	Vineyard: grape	36.634	119.768	77	77	1.00	0.000		Fresno	1461
K2	Vineyard: grape	36.592	119.764	51	26	0.58	0.376		Fresno	1461
K3	Vineyard: grape	36.620	119.777	75	70	0.94	0.056		Fresno	1461
L1	Vineyard: grape	35.906	119.250	60	60	1.00	0.000		Tulare	1609
L2	Vineyard: grape	35.906	119.224	60	60	1.00	0.000		Tulare	1609
N1	Vineyard: grape	35.703	119.391	51	50	0.98	0.024		Kern	42
CSU	Orchard: peach	36.818	119.734	–	–	–	–		Fresno	1461
WES	Vineyard: grape	36.340	120.106	–	–	–	–		Fresno	1461
MCC	Vineyard: grape	36.638	119.611	–	–	–	–		Fresno	1461
KEA	Vineyard: grape	36.595	119.507	–	–	–	–		Fresno	1461
Nonagricultural habitats										
MEN2	Roadside	39.179	123.683	60	0	0.00	0.000	0.07	Mendocino	3
AUB1	Roadside	38.972	121.106	90	3	0.03	0.019		Placer	140
YOL1	Roadside	38.938	122.225	51	2	0.04	0.007		Yolo	78
MEN1	Roadside	38.883	123.141	27	9	0.33	–		Mendocino	3
SAC1	Roadside	38.486	121.081	60	2	0.03	0.033		Sacramento	395
NAP1	Roadside	38.483	122.240	57	0	0.00	0.000		Napa	0
BOD1	Roadside	38.306	123.058	47	0	0.00	0.000		Sonoma	36
BOD2	Roadside	38.306	122.839	67	13	0.14	0.128		Sonoma	36
LIV1	Roadside	37.703	121.737	60	1	0.02	0.017		Alameda	0
MAR	Roadside	37.483	119.963	59	1	0.02	0.017		Mariposa	0
CRU1	Natural reserve	36.950	122.065	54	4	0.07	0.069		Santa Cruz	0
MON2	Roadside	36.436	121.630	90	16	0.18	0.048		Monterey	156
Control populations										
R	Canal bank	36.488	119.403	193	185	0.96	0.022		Tulare	1609
S	Orchard	36.799	119.954	255	10	0.02	0.023		Fresno	1461

R, population‐level resistance to glyphosate estimated as the proportion of survivors out of the total number of plants treated with glyphosate at 840 g a.e. ha^−1^, averaged over 2–3 replications.

Groundwater protection area (GWPA) per county available at http://www.cdpr.ca.gov/docs/emon/grndwtr/gwpa_lists.htm.

Resistant (R) and susceptible (S) controls from Shrestha et al. (2007).

### Analysis of response to glyphosate

Equal amounts of seed from each plant, by volume, were bulked to produce a seed sample for each of the 38 populations. The bulked population seed samples were germinated on the surface of moist soil in 30‐cm‐diameter plastic pots. Young seedlings were transplanted into 5 × 5 cm square pots with one seedling per pot. Modified UC soil mix (peat, sand, and redwood compost in 1:1:1 ratio) was used. Plants were watered almost daily as needed and fertilized biweekly with Hoagland solution. In total, 40–90 plants were tested for glyphosate response with 2–3 replications per population and up to 30 plants per replication with the exception of population MEN1, which had one replication of 27 plants due to mortality (Table 1). For every replication, 1–3 susceptible and resistant control plants from previously characterized seed lots (Shrestha et al. 2007) were included as controls. All plants were grown in a single greenhouse under ambient light conditions between April and October 2011 in Davis, California, USA. Plants were sprayed at the five‐ to eight‐leaf stage using a track sprayer (Technical Machinery Inc., Sacramento, CA, USA) at the label rate of acid equivalent 840 g ha^−1^ of glyphosate (Roundup WeatherMax; Monsanto Company, St. Louis, MO, USA) in a spray volume of 140 L ha^−1^ with deionized water as the carrier at 207 kPa. Response to glyphosate was assessed visually for each plant 35 days after glyphosate treatment and characterized at the population level by the proportion of plants surviving glyphosate treatment of the total number of plants treated per replication and averaged over replications.

Each of the areas designated as GWPA is 2.6 km^2^ of land that is vulnerable to the movement of pesticides into ground water by either leaching or runoff processes (CADPR 2004; http://www.cdpr.ca.gov/docs/emon/grndwtr/gwpa_locations.htm). The total area designated as GWPA within counties was compiled from the list available at http://www.cdpr.ca.gov/docs/emon/grndwtr/gwpa_lists.htm. To test for correlation between frequency of resistant plants within populations and the size of GWPA within counties in which the populations were sampled, the cor.test function based on Pearson's product‐moment correlation was used in R 2.14.2 (R Development Core Team 2011).

### Microsatellite analysis

DNA was extracted from dried leaf tissue using the CTAB procedure (Doyle and Doyle 1987). Individuals were genotyped using 12 microsatellite markers, which included HW02, HW06, HW07, HW14, HW29 (Molecular Ecology Resources Primer Development Consortium 2009), HW17 (GenBank: EU652944.1), and six additional new microsatellite markers (Table S1; HWSSR01, HWSSR03, HWSSR04, HWSSR11, HWSSR09, and HWSSR12). Primer sequences for the six new markers were developed using the assembled contigs from 454 sequencing of the *C. canadensis* genomic DNA samples (Y. Peng, Z. Lai, T. Lane, M. Okada, M. Jasieniuk, L. Rieseberg and C.N. Stewart, Jr., unpublished data). Contigs containing microsatellites were identified using the Simple Sequence Repeat Identification Tool (SSRIT, Temnykh et al. 2001, http://www.gramene.org/db/markers/ssrtool). Primers flanking the microsatellite sequences were designed using Primer Premier 5.0 (Premier Biosoft, Palo Alto, CA, USA). Polymerase chain reactions (PCR) were performed in multiplex of three microsatellite markers in a total volume of 5 μL containing 10 ng of template DNA, 0.6–0.8 μm each of fluorescence‐labeled forward primer and unlabeled reverse primer, 125 μm dNTPs, 0.375 unit of *Taq* polymerase (QIAGEN, Valencia, CA, USA), and 1× PCR buffer (QIAGEN) using 384‐well PCR plates. PCR products were separated and sized on an ABI Prism 3100 Genetic Analyser (Applied Biosystems, Foster City, CA, USA) with GENEMAPPER version 3.7 using GENESCAN 500 ROX for markers HW02, HW29, and HWSSR09 and GENESCAN 400HD ROX for all other markers as internal size standard. A total of 13 individuals (one plant in population A2, two in B2, seven in C1, one in G2, one in I2, and one in MEN2) exhibited allelic profiles of a polyploid similar to the often co‐occurring hexaploid *Conyza bonariensis* (2*n* = 6*x* = 54), obviously in addition to the diploid *C. canadensis* alleles (2*n* = 2*x* = 18) in some cases, suggesting rare incidences of interspecific hybridization. However, for the purposes of the analyses of population genetic diversity and structure in *C. canadensis* in this study, the 13 individuals were excluded from analyses following re‐analysis of the original leaf samples verifying the genotyping results.

### Molecular data analyses

#### Genetic diversity and structure

To estimate genetic diversity within loci, the total number of alleles detected (*T*_A_), expected heterozygosity (*H*_E_), observed heterozygosity (*H*_O_), and Weir and Cockerham's (1984) estimation of Wright's inbreeding coefficient (*F*_IS_) and fixation index (*F*_ST_) were calculated for each locus using FSTAT 2.9.3 (Goudet 2002). Statistical significance of the *F*_IS_ and *F*_ST_ values was determined with 1000 randomizations. Random mating was not assumed for *F*_ST_. To estimate genetic diversity within populations over the 12 microsatellite loci, allelic richness (*A*), *H*_E_, *H*_O_, *F*_IS_ were calculated for each population using FSTAT 2.9.3. To assess the pattern of mating in populations, the rate of self‐fertilization (*s*) was estimated as 2*F*_IS_/(1 +* F*_IS_). To test for correlations between resistance and genetic diversity measures or selfing rates, the cor.test function based on Pearson's product‐moment correlation was used in R 2.14.2 (R Development Core Team 2011).

Genetic differentiation among sampled populations was assessed by calculating pairwise *F*_ST_ values between all pairs of sampled populations using FSTAT 2.9.3. Statistical significance of the *F*_ST_ values was assessed using 1000 permutations with the Bonferroni procedure to correct for multiple tests. To elucidate geographical structuring of genetic variation among populations, a distance‐based clustering analysis was used. Nei's genetic distances (Nei 1978) were computed between all pairs of populations with 1000 bootstrap replications using MICROSATELLITE ANALYSER (Dieringer and Schlötterer 2003). The program FITCH in PHYLIP version 3.57c (Felsenstein 2005) was used to construct the dendrogram based on the Fitch–Margoliash least squares method with branch lengths inferred using the consensus tree as user tree.

To assess the spatial patterns of seed dispersal (Siol et al. 2008), the distribution of shared multilocus genotypes (MLGs) among populations was analyzed. All shared MLGs among all individuals were identified using MICROSATELLITE TOOLKIT (Park 2001). Then, for only the MLGs shared by multiple populations, the number of each MLG was compiled per population. To gain insight into the number of independent origins of glyphosate resistance, MLGs in highly resistant populations were classified as nonrecombinant or recombinant with respect to other MLGs within the populations (Siol et al. 2008).

Population structure was further investigated using the model‐based Bayesian clustering program InStruct (Gao et al. 2007). The analytical approach used in InStruct is based on the widely used program STRUCTURE (Pritchard et al. 2000) and was developed for self‐fertilizing organisms to overcome the problem of spurious population structure or admixture that may be detected when Hardy–Weinberg equilibrium is assumed when analyzing selfing populations (Falush et al. 2003; Gao et al. 2007). To determine *K*, the number of populations, or gene pools, analyses were conducted for *K* values ranging from 1 to 42 using MCMC iterations of 1 000 000 with a burn‐in period of 500 000, thinning interval of 100, and a posterior credible interval of 0.95 for five chains at each *K*. ln *P*(*D*) (Pritchard et al. 2000) and *ΔK* (Evanno et al. 2005) were used to infer *K*. The programs CLUMPP (Jokobsson and Rosenberg 2007) and DISTRUCT (Rosenberg 2004) were used to assess the extent of multimodality or substantially different clustering solutions among runs (Pritchard et al. 2010). Multimodality is a characteristic of data sets with complex relationship among individuals within a data set with a relatively large *K* (Rosenberg et al. 2001; Pritchard et al. 2010).

#### Approximate Bayesian computation (ABC) analysis

**Figure 2:**
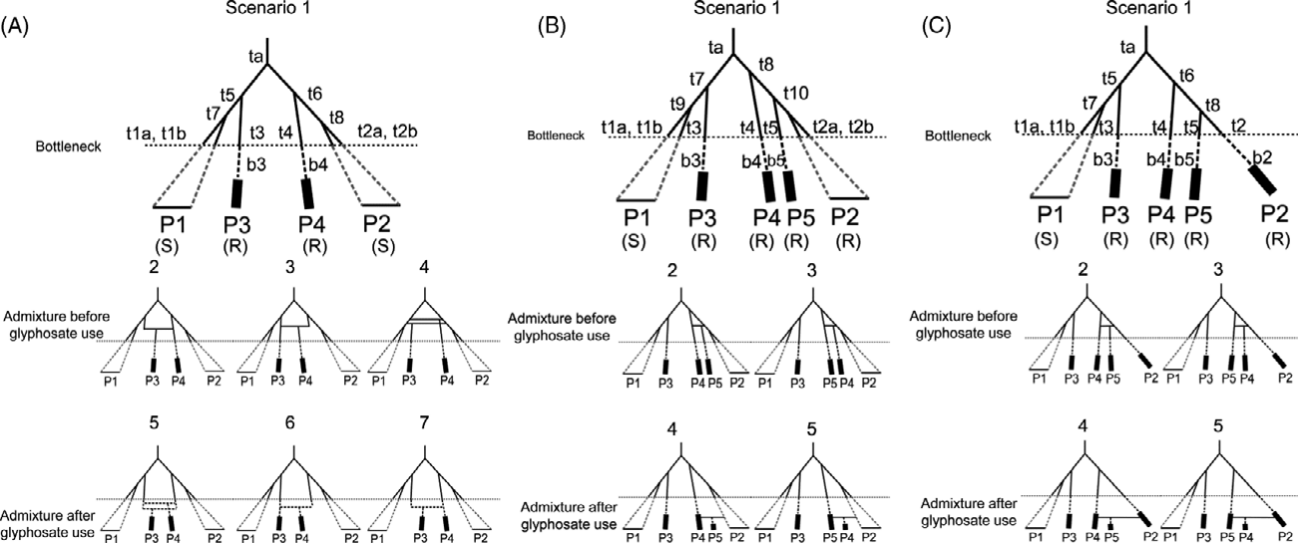
Three sets of scenarios used in the DIYABC analyses (Cornuet et al. 2008) of *Conyza canadensis* populations. (A) Set of seven scenarios: Scenarios 1 through 4 model independent origins of glyphosate resistance in P3 and P4 by the absence of admixture or the occurrence of admixture prior to glyphosate selection; and Scenarios 5 through 7 model common origins by recent admixture event(s) prior to population expansion in lineages leading to resistant populations. (B) Set of five scenarios: Scenarios 1 through 3 model independent origins of glyphosate resistance in P3, P4, and P5 by the absence of admixture or the occurrence of admixture prior to glyphosate selection. Scenarios 4 and 5 model common origins by recent admixture event(s) prior to population expansion. (C) Set of five scenarios: Scenarios 1 through 3 model independent origins of glyphosate resistance in P2, P3, P4, and P5 by the absence of admixture or the occurrence of admixture prior to glyphosate selection and Scenarios 4 and 5 model common origins by recent admixture event(s) prior to population expansion. All scenarios model two groups of populations sampled at present and diverged from a single population in the past at time step, *ta*, corresponding to the two gene pools at *K *=* *2 identified at the highest hierarchical level of population structure in the Bayesian clustering analysis. Variations in line patterns and width in the branches of the coalescent tree indicate possible effective population size changes: thin solid line for prior to agricultural weed management, *N*_*e*_; dashed line during bottleneck, *N*_*b*_; thick line for expanded resistant population, *N*_*r*_. All lineages are assumed to have undergone reductions in effective population size, indicated by the dashed horizontal line. Glyphosate‐resistant populations undergo population expansion after a bottleneck at time *t*3−*b*3, *t*4−*b*4, and/or *t*5−*b*5. In contrast, glyphosate‐susceptible populations remain in bottleneck until present. Based on the ‘quality of scenario/prior combinations’ analysis in DIYABC, two lineages had greater potential to generate simulated data closer to the observed data for susceptible populations than a single lineage.

To examine whether resistance to glyphosate became widespread following a single or multiple origins, we conducted approximate Bayesian computation (ABC) analyses using the program DIYABC (Cornuet et al. 2008). Three sets (A, B, and C) of scenarios hypothesizing the evolutionary and demographic histories of susceptible and resistant populations were evaluated (Fig. 2A–C). For set A (Fig. 2A), the seven scenarios were tested using two glyphosate‐resistant and two glyphosate‐susceptible populations chosen from the two major gene pools identified at *K *=* *2 by INSTRUCT analysis. Scenarios 1, 2, 3, and 4 were characterized by the absence of recent admixture (hybridization) in the history of the resistant populations. Scenarios 2, 3, and 4 included historical admixture events prior to the use of glyphosate in agriculture. Scenarios 5, 6, and 7 modeled recent admixture, that is, after glyphosate came into use. Recent admixture since the herbicide glyphosate came into use could have led to spread of resistance from one lineage to another following a single origin of resistance. Alternatively, support for scenarios with no such recent admixture indicates independent origins of resistance. For sets B (Fig. 2B) and C (Fig. 2C), five scenarios were tested using three glyphosate‐resistant and two glyphosate‐susceptible populations (Fig. 2B) and four glyphosate‐resistant populations and one glyphosate‐susceptible population (Fig. 2C). Scenarios 1, 2, and 3 modeled independent origins of resistance, while Scenarios 4 and 5 modeled spread of resistance by admixture.

To estimate the timing of changes in *N*_*e*_ in lineages leading to resistant and susceptible populations, the start of the bottleneck or reduction in *N*_*e*_ was modeled as a time step in generations before the present, in each of the lineages. For resistant populations P3, P4, and P5, the timing of population expansion due to evolution of glyphosate resistance was modeled as the start of bottleneck minus the duration of bottleneck (i.e., *t*3*−b*3, *t*4*−b*4, *t*5*−b*5, respectively, and *t*3* > b*3, *t*4* > b*4, and *t*5* > b*5). The susceptible populations were modeled to remain in a state of bottleneck to the present. Because of the annual life cycle, the number of generations should equal the number of years.

Glyphosate has been commercially available since 1974 (Duke and Powels 2008); thus, the prior distributions for the timing of the recent admixture were bound between 1 and 40 generations ago, after the advent of glyphosate use in the environment. Priors for timing of the population bottleneck in each lineage were set to range from 1 to 150 generations ago, to include the start of intensive and extensive agriculture in California around 1890 (Johnston and McCalla 2004), hence, the possible effects of tillage and other herbicides that appeared to have kept the populations in check prior to intensive use of glyphosate, at least in the southern Central Valley (K. Hembree unpublished data; S. Wright unpublished data). The priors for divergence times since the most recent common ancestor of all lineages were set to range from the time step before the bottleneck to the default value of 10 000. Default priors were used for effective population size ranging from 10 to 10 000 with the exception of all bottleneck effective population sizes and the resistant, expanded effective population size, which were 2–100 and 10–100 000, respectively. The priors for all admixture rates were the default values ranging from 0.001 to 0.999. Because of the highly self‐fertilizing mating system, priors for mutation rates were initially scaled by 1/2 of default values (Nordborg 2000). The probability distributions of all priors were the program's default settings.

To check whether simulated data sets based on the prior distributions and the models can potentially generate summary statistics close to the observed data, 10 000 simulations per scenario were performed by using the option ‘quality of scenario/prior combinations’ in DIYABC (Cornuet et al. 2010). Based on these ‘first shot’ simulations (Bertorelle et al. 2010), mutation rates (10^−1^ of the program default values) and mean coefficient *P* (geometric distribution in the generalized stepwise mutation model; 0.100–0.70) were chosen. In addition, susceptible populations were modeled as consisting of two closely related but divergent lineages from the same cluster in all scenarios. The model of multiple lineages within a population is consistent with high allelic and genotypic diversity despite high selfing rates in many susceptible populations.

The within‐population summary statistics used in the ABC analysis (Cornuet et al. 2008) were the mean number of alleles and the mean coefficient *M*, the ratio of the number of alleles to the range in allele size (Garza and Williamson 2001), which are informative for changes in effective population size. The coefficient *M* is also sensitive to extreme population subdivision that could result from the self‐fertilizing mating system and presence of multiple distinct lineages within a population. For all pairs of populations, *F*_ST_ (Weir and Cockerham 1984) and mean individual assignment likelihood (Rannala and Mountain 1997; Pascual et al. 2007), informative for population divergence and admixture, were used.

One million simulations were conducted per scenario. Scenarios were compared by estimating their posterior probabilities using local logistic regression on 70 000 and 50 000 best simulations for seven and five scenarios, respectively. Power analyses were conducted by estimating Type I and Type II error for each scenario using the ‘confidence in scenario choice’ option based on 500 simulated data sets per scenario. Parameter estimates were obtained for the selected scenarios, and to assess the goodness‐of‐fit of the scenarios to the data, 10 000 data sets were simulated with parameter values from the posterior distribution using the ‘estimate posterior distribution of parameters and model checking’ option. Three test statistics, including mean genic diversity (Nei 1987) and mean allele size variance within populations and shared allele distance (Chakraborty and Jin 1993) between populations, were used to compare the observed data to the simulated data. The three test statistics selected for model checking were not used in scenario selection and parameter estimation to avoid over estimating goodness of fit (Cornuet et al. 2010). *P*‐values were adjusted for multiple tests using the p.adjust function in R 2.14.2 (R Development Core Team 2011) based on the false discovery rate correction method of Benjamini and Hochberg (1995).

## Results

### Glyphosate resistance

**Figure 3:**
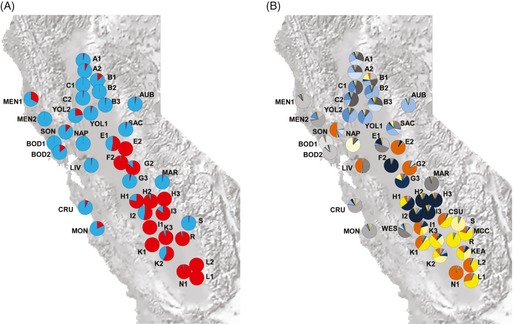
Map of *Conyza canadensis* populations, (A) response to glyphosate in each population as proportion of resistant (red) and susceptible (light blue) plants based on greenhouse screening of plants grown from field collected seeds, and (B) average assignment per population at *K *=* *14 in the run with the highest probability of the data (InStruct, Gao et al. 2007).

Population‐level resistance to glyphosate, estimated as the proportion of survivors of the total number of plants treated with glyphosate per population, varied from 0 to 1.00 among populations and was strongly spatially structured (Table 1; Fig. 3A). Average frequencies of resistant plants within populations from the northern, central, and southern regions of the Central Valley were 0.07, 0.68, and 0.88, respectively (Table 1). Northern populations had substantially lower frequencies of resistant plants than central or southern populations. Populations sampled in nonagricultural human‐disturbed habitats, which were mostly roadsides, were largely susceptible to glyphosate. Areas designated as GWPA within the 19 counties where populations were sampled varied between zero to 1609 km^2^ (Table 1). The frequency of herbicide‐resistant plants within populations was significantly positively correlated (*r*_38_ = 0.628, *P *=* *1.41 × 10^−5^) to the area in km^2^ of the GWPA within each county in which populations were sampled based on Pearson's product‐moment correlation.

### Genetic diversity and spatial structure

A total of 125 alleles were detected over the 12 microsatellite loci with the number of alleles ranging from 2 to 23 per locus, indicating that the loci were highly variable and informative (Table S2). Observed heterozygosity (*H*_O_) within populations ranged from 0.000 to 0.024, whereas expected heterozygosity (*H*_S_) ranged from 0.003 to 0.445. Accordingly, inbreeding coefficients (*F*_IS_) ranged from 0.850 to 0.966 over loci and were highly significant, consistent with the highly selfing mating system. Expected heterozygosity (*H*_T_) over all samples ranged from 0.003 to 0.894 over loci, and *F*_ST_ ranged from 0.034 to 0.753 and indicated significant differentiation among populations at all loci.

Within populations, allelic richness (*A*) ranged from 1.0 to 4.3 among populations (Table 2). Expected heterozygosity (*H*_E_) varied from 0.00 to 0.45 among populations, whereas observed heterozygosity (*H*_O_) ranged from 0.00 to 0.10. No heterozygous individuals were observed in 20 of the 44 populations. Inbreeding coefficients (*F*_IS_) were uniformly high and significant in all populations, indicating a highly selfing mating system, with the exception of population F2 (Table 2). Population F2 consisted of a single MLG with the exception of one individual that was heterozygous at a single locus because of the presence of an allele with a mutation consisting of an additional single microsatellite repeat unit. Detection of a rare mutant allele might indicate recent population expansion consistent with recent colonization or spread of resistance, not unusual for weed populations. For the purpose of estimating *s*,* H*_O_ was considered to be zero in this population. Estimates of selfing rate, *s*, based on *F*_IS_ (Allard et al. 1968) ranged from 0.772 to 1.000 with an average over all 35 polymorphic populations of 0.964. Within‐population genetic diversity measures (*A*,* H*_E_, *H*_O_) and selfing rates (*s*) were not significantly correlated with the frequency of glyphosate‐resistant plants over all sampled populations (*r*_38_ = −0.082, *P *=* *0.615; *r*_38_ = −0.122, *P *=* *0.454; *r*_38_ = −0.173, *P *=* *0.286; *r*_33_ = 0.133, *P *=* *0.446, respectively) or over all orchard or vineyard populations (*r*_25_ = −0.231, *P *=* *0.247; *r*_25_ = −0.201, *P *=* *0.315; *r*_25_ = −0.235, *P *=* *0.237; *r*_22_ = 0.036, *P *=* *0.866, respectively) based on Pearson's product‐moment correlation.

**Table 2:** Genetic diversity and selfing rate estimates within populations of *Conyza canadensis*

ID	*n*	*A*	*H*_E_	*H*_O_	*F*_IS_	*s*
Northern region						
A1	30	2.5	0.33	0.06	0.837	0.911
A2	28	3	0.33	0.01	0.978	0.989
B1	30	3.7	0.37	0	1	1
B2	28	2.1	0.19	0	1	1
B3	30	1.7	0.2	0.02	0.914	0.955
C1	23	2.1	0.19	0.01	0.979	0.989
C2	29	2.4	0.25	0	1	1
YOL2	30	2.3	0.21	0.01	0.712	0.832
SON1	30	2.7	0.42	0.04	0.875	0.933
Central region						
E1	29	1.6	0.04	0	1	1
E2	30	2.2	0.28	0.01	0.952	0.976
F2	30	1.1	0	0	−0.009^ns^	−0.017
G2	27	2.2	0.15	0	1	1
G3	30	2.4	0.12	0	1	1
Southern region						
H1	30	2.9	0.34	0	1	1
H2	30	1.9	0.06	0	1	1
H3	30	1.9	0.06	0	1	1
I1	30	2.8	0.4	0	0.994	0.997
I2	29	2.1	0.17	0.01	0.969	0.984
I3	30	2	0.08	0.01	0.947	0.973
K1	30	2.2	0.32	0.02	0.954	0.976
K2	30	2.2	0.31	0.02	0.947	0.973
K3	28	2.2	0.31	0.01	0.977	0.988
L1	30	2.4	0.3	0.02	0.942	0.97
L2	30	4.3	0.45	0.01	0.987	0.994
N1	30	2.6	0.31	0.03	0.817	0.9
CSU	30	1.8	0.27	0.02	0.918	0.957
WES	30	2.1	0.35	0.1	0.685	0.813
MCC	30	1.8	0.24	0	0.97	0.985
KEA	30	1	0	0	–	–
Nonagricultural habitats						
MEN2	29	2.4	0.23	0.02	0.906	0.951
AUB1	30	1.4	0.11	0	1	1
YOL1	30	2.2	0.22	0	0.991	0.995
MEN1	30	1.3	0.02	0	0.831	0.908
SAC1	30	1.9	0.2	0.01	0.778	0.875
NAP1	30	1.8	0.08	0.01	0.933	0.965
BOD1	30	1	0	0	–	–
BOD2	30	2.6	0.32	0	0.982	0.991
LIV1	30	1.9	0.35	0.01	0.983	0.991
MAR	30	1.6	0.11	0.01	0.906	0.95
CRU1	30	1.9	0.24	0.01	0.973	0.986
MON2	30	1.9	0.16	0.02	0.629	0.773
Control populations						
R	30	1	0	0	–	–
S	30	1	0	0	–	–

*n*, sample size; *A*, mean allelic richness; *H*_E_, mean expected heterozygosity; *H*_O_, mean observed heterozygosity; *F*_IS_, mean inbreeding coefficient across 12 microsatellite loci; *s*, selfing rate estimated as 2 *F*_IS_/(*F*_IS_ + 1).

Populations are sorted by latitude and habitat type.

ns, not significant at α = 0.05 after Bonferroni correction.

R and S control populations from Shrestha et al. (2007).

**Figure 4:**
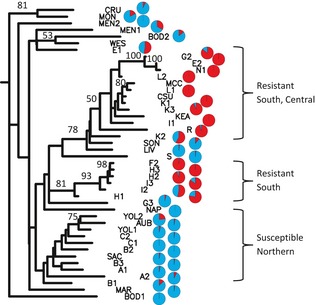
A dendrogram on pairwise distances between *Conyza canadensis* populations based on the Fitch‐Margoliash method. Bootstrap values >50% at nodes are indicated. Population‐level glyphosate resistance is shown as average proportion of survivors (red) over total treated as pie charts.

The clustering of populations based on pairwise genetic distances identified two groups of predominantly resistant populations with bootstrap support >50% (Fig. 4) in vineyards from the central and southern areas and in orchards from the central part of the Central Valley, indicating extensive spread of resistance in these areas. Remaining populations were predominantly susceptible and from the northernmost part of the valley with no substantial genetic structuring among them.

Pairwise *F*_ST_ estimates between populations ranged from 0.00 to 1.00 (Table S3). Most populations were highly differentiated with 946 significant pairwise *F*_ST_ values between populations of the 986 tested, as expected for a highly selfing species (Hamrick and Godt 1996; Charlesworth 2003). Population pairs with nonsignificant *F*_ST_ formed two groups of populations: E2, G2, L2, N1, LIV, SON, I1, K1, K3, L1, L2, CSU, and MCC; and F2 H2, H3, I2, I3, H1, H2, and H3. The two groups corresponded to the two well‐supported groups of predominantly resistant populations in the clustering analysis of populations (Fig. 4), also indicating the spread of resistance among populations within the two groups.

A total of 272 unique MLGs were found among the 1300 individuals genotyped at the 12 microsatellite loci. Of the 272 genotypes, 108 were found multiple times and made up 79% of individuals sampled. Of the 108 genotypes, 35 were shared among populations (Figure S1A) and accounted for 50% of all plants sampled. Two of the 35 were prominently widespread, occurring 159 and 151 times among the 1300 individuals, and in 15 and six populations, respectively (Figure S1B). The distribution of the 35 MLG among populations corresponded to the three groups shown in Fig. 4, indicating dispersal predominantly by seed in both resistant and susceptible populations.

A total of 35 unique MLGs were found within nine highly resistant (*R* ≥ 0.95) populations, E2, F2, H2, H3, I1, K1, L1, L2, and N1 (Tables 3 and S4). MLGs b and c are possibly the same selfing lineage differing only by a single‐repeat‐unit microsatellite allele that is otherwise absent in the population. MLG a and the singleton in population F2, and MLG c and the singleton in population I1 are also likely to be the same selfing lineage differing only by a single mutation. The presence of multiple distinct selfing lineages (e.g., MLG a, b, d, e, and f) in the highly resistant populations suggests that resistance evolved independently multiple times in this species. Of the 35 MLG detected in highly resistant populations, 13 may be recombinant with respect to other more abundant MLGs within the populations (Tables 3 and S4). All 13 potentially recombinant MLGs, which suggest outcrossing and possibly spread of resistance by pollen within populations, were detected in populations K1 and L1.

**Table 3:** Numbers of distinct multilocus genotypes (MLGs) within *Conyza canadensis* populations with frequencies of glyphosate‐resistant individuals (*R*) of ≥0.95. MLGs that appear recombinant with respect to other more abundant genotypes within the population are indicated by bold numbers. Genetic cluster(s) from Bayesian clustering at *K *=* *14 (Figs 3B and 5B) to which MLGs assign highly are indicated where D, dark blue; O, orange; Y, yellow, L, light yellow; B, blue

Population	Multilocus genotypes													
	a*	b†	c†	d	e	f	g	h	i	j	k	l	m	Singletons
E2		28												1
F2	29													1*
H2	27													3
H3	28	1												1
I1	1		14		10	2				2				1*
K1		10				1	**1**	**5**	**3**		**1**			**9** a
L1		6		8		7	**1**				**1**	**2**		**5** a
L2		18		3			**6**						2	1
N1		30												0
Total	85	93	14	11	10	10	8	5	3	2	2	2	2	
Cluster	D	O	O	Y	L/B	Y	Y	Y	Y	Y/O	Y	O	Y/L	

*,†MLGs that are possibly the same selfing lineage differing only by a single‐repeat‐unit microsatellite allele that is otherwise absent in the population.

a Five of the nine and three of the five unique genotypes in populations K1 and L1, respectively, were likely recombinant.

**Figure 5:**
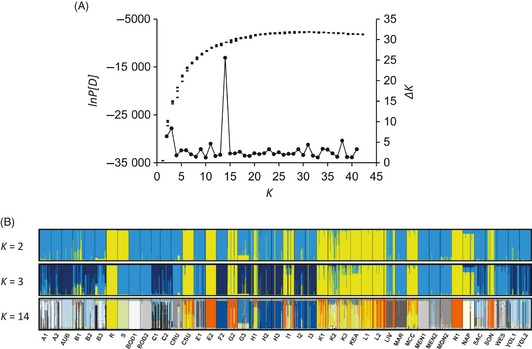
Bayesian clustering analysis (INSTRUCT, Gao et al. 2007) of *Conyza canadensis* (A) plot of the log likelihood of the multilocus genotypic data, ln *P*(*D*), for five runs at each value of *K*, and the second order rate of change in ln *P*(*D*), Δ*K*, as a function of the number of clusters or gene pools, *K,* and (B) probabilities of assignment of individuals at *K *=* *2, *K *=* *3, and *K *=* *14 in the run with the highest probability of the data. Each vertical bar represents an individual and the proportion of its genome that assigned to distinct clusters.

InStruct analysis (Gao et al. 2007) revealed gradually increasing values of ln *P*(*D*), but no clear maximum, with increasing *K* values ranging from 1 to 42 (Fig. 5A), indicating a hierarchical pattern of population structure (Evanno et al. 2005). Δ*K* (Evanno et al. 2005) showed the highest peak at *K *=* *14, followed by peaks at *K* = 3 and *K* = 2. The 14 genetic clusters or gene pools were grouped into two genetic clusters at the uppermost hierarchical level of genetic structuring at *K *=* *2 and into three clusters at *K *=* *3 (Fig. 5B). Gelman‐Rubin statistics, which test for convergence, were <1.10 at all *K* indicating convergence among runs (Gao et al. 2007). However, over the five runs at *K *=* *2, 3, and 14, multimodality in assignment to distinct clusters was apparent in many individuals. The run with the highest ln *P*(*D*) was not the most frequent clustering solution within five runs for *K *=* *3 (data not shown). Multimodality in the assignment in many individuals among runs persisted with increased MCMC replications of 2 000 000 with a 1 000 000 burn‐in period (data not shown).

The two genetic clusters identified at *K *=* *2 in the INSTRUCT analysis were spatially structured into northern and southern gene pools or clusters as apparent in the spatial distribution of the subclusters at *K *=* *14 (Figs 3B and 5B). The geographical distribution of the northern gene pool (shades of blue and grey) ranged from the northernmost sampling region A to the sampling region I, whereas distribution of the southern gene pool (shades of orange and yellow) was mainly concentrated in the southern most sampling areas I through L with some presence in the central part of the sampled range. Most of the populations in the southern areas of the valley were predominantly resistant and assigned to both gene pools, but the northernmost populations in sampling regions A, B, C were predominantly susceptible and assigned to the northern gene pool (Figs 3A,B and 5B). At *K *=* *14, highly resistant populations with *R* ≥ 0.95 assigned mostly to three of the 14 clusters, suggesting that there were three likely independent origins of the resistance trait. One of the three was from the northern gene pool and two of the three were from the southern gene pool. Predominantly susceptible populations assigned mainly to the remaining 11 gene pools.

### Approximate Bayesian computation analysis

Because DIYABC analyses are computationally intensive, we selected a subset of populations and MLGs to analyze using this method. Resistant populations and MLGs included the highly resistant (*R* ≥ 0.95) populations, F2 and N1 (Table 1), that essentially consisted of resistant MLGs, a and b, respectively, as well as seven additional resistant MLGs, c, d, e, f, h, i, and k (Table 3). For analysis of resistant populations, MLGs were used because in a highly selfing species, MLGs represent evolutionary lineages, whereas sampled populations may consist of distantly related lineages with distinct population histories. Because the evolutionary history of each MLG can be modeled as a single lineage, the use of MLGs in the analyses simplified scenario sets and simulations and thus allowed simplified analyses of multiple resistant MLGs in multiple analyses. On the other hand, for the susceptible populations, we used C2 (Table 1) as population P1 in the scenarios shown in Fig. 2A–C and population S (Table 1) as population P2 in the scenarios shown in Fig. 2A,B. Susceptible populations C2 and S assigned to the northern and southern gene pools, respectively, were identified by INSTRUCT analysis at *K *=* *2 (Fig. 5B).

For scenario sets A and B (Fig. 2A,B), we investigated whether resistance evolved independently or had common origins, among the three genetic clusters, to which most individuals in highly resistant populations assigned, in Bayesian clustering at *K* = 14 (Fig. 5A,B). Resistant MLGs a, b, and d were used to represent the three genetic clusters (Table 3). For scenario set A (Fig. 2A), we selected population F2, consisting essentially of MLG a (dark blue cluster in Fig. 3B), as P3 (Fig. 2A), and population N1, consisting of MLG b (orange cluster in Fig. 3B), as P4 (Fig. 2A). Subsequently, for scenario set B (Fig. 2B), independent or common origin was tested between MLG d (yellow cluster in Fig. 3B) as P5 and MLG b as P4, while MLG a was used as P3.

One of the analyses using scenario set C (Fig. 2C) investigated independent or common origins among additional MLGs that were assessed to be nonrecombinant, including MLGs a, c, e, and f and that assigned to distinct clusters in Bayesian clustering at *K* = 14 (Table 3). The resistant MLGs a, c, e, and f were used as P3, P2, P4, and P5 (Fig. 2C), respectively. Two additional analyses of scenario set C (Fig. 2C) were conducted using recombinant resistant MLGs that are expected to have common origins with other resistant MLGs. One analysis used resistant MLGs a, b, d, and k and the other analysis used resistant MLGs a, b, h, and i, as P3, P2, P4, and P5 (Fig. 2C).

For the analysis based on scenario set A (Fig. 2A), Scenario 1 with no admixture was the most highly supported but had relatively weak posterior probability of 0.35 (Table 4). Similarly, in the analysis of scenario set B (Fig. 2B), Scenarios 1 and 2 with no admixture and historical admixture, respectively, were most highly supported. Also, analysis of scenario set C (Fig. 2C) with nonrecombinant, resistant MLGs c, a, e, and f (Table 3) revealed that Scenario 2 with historical admixture was the most highly supported. In the above three analyses, all the scenarios with no recent admixture since the use of glyphosate, that is, Scenarios 1 through 4 together (Fig. 2A) and Scenarios 1 through 3 together (Fig. 2B,C), had high posterior probabilities of 0.98, 0.99, and 1.00, providing strong support for independent origins of resistance in MLGs a (population F2), b (population N1), d, e, and f. In contrast, in the two analyses of scenario set C using recombinant MLGs, Scenario 4 with recent admixture was the most highly supported with strong posterior probabilities of 0.86 and 0.84 (Table 4), providing support for possible spread of resistance by pollen although independent recent origins of resistance cannot be ruled out.

**Table 4:** Posterior probabilities and associated 95% confidence intervals of scenarios that model the population history of glyphosate‐resistant and glyphosate‐susceptible populations of *Conyza canadensis*, based on the logistic estimate obtained in an ABC analysis using DIYABC (Cornuet et al. 2008). Logistic regression was performed on the 70 000 and 50 000 simulations closest to the observed value for analyses with seven scenarios and five scenarios, respectively. Populations (Table 1) and multilocus genotypes (Table 3) used in the analyses are listed in order from P1 through P4 for scenario set A (Fig. 2A) or P1 through P5 for scenario sets B and C (Fig. 2B,C). The posterior probabilities for the most supported scenarios are in bold numbers

Scenario	Admixture in the scenario	Posterior probability	95% confidence interval
A: C2, S, F2(a), N1(b)			
1	None	**0.3542**	0.3251, 0.3832
2	Historical	0.1954	0.1742, 0.2166
3	Historical	0.1895	0.1700, 0.2091
4	Historical	0.2444	0.2204, 0.2683
5	Recent	0.0010	0.0001, 0.0019
6	Recent	0.0063	0.0035, 0.0091
7	Recent	0.0092	0.0052, 0.0133
B: C2, S, a, b, d			
1	None	**0.3946**	0.3542, 0.4350
2	Historical	**0.3929**	0.3534, 0.4323
3	Historical	0.2018	0.1741, 0.2296
4	Recent	0.0079	0.0036, 0.0122
5	Recent	0.0027	0.0008, 0.0047
C: C2, c, a, e, f			
1	None	0.1705	0.1469, 0.1941
2	Historical	**0.5050**	0.4673, 0.5427
3	Historical	0.3212	0.2870, 0.3553
4	Recent	0.0033	0.0006, 0.0061
5	Recent	0.0000	0.0000, 0.0001
C: C2, b, a, d, k			
1	None	0.0114	0.0071, 0.0157
2	Historical	0.1053	0.0688, 0.1419
3	Historical	0.0195	0.0123, 0.0267
4	Recent	**0.8636**	0.8169, 0.9103
5	Recent	0.0001	0.0000, 0.0002
C: C2, b, a, h, i			
1	None	0.0277	0.0000, 0.0656
2	Historical	0.1148	0.0000, 0.2653
3	Historical	0.0178	0.0000, 0.0423
4	Recent	**0.8396**	0.6316, 1.0000
5	Recent	0.0000	0.0000, 0.0000

**Table 5:** Type I and Type II error rates when each scenario, modeling the population history of glyphosate‐resistant and glyphosate‐susceptible populations of *Conyza canadensis*, was used to simulate data in DIYABC (Cornuet et al. 2010). The most likely scenario selected for the simulated data and Type I and Type II error rates for no recent admixture (Scenarios 1–4 or 1–3) versus recent admixture (Scenarios 5–7 or 4–5)

	True scenario used for simulation							Type I error rate		Type II error rate	
	1	2	3	4	5	6	7				
Frequency of scenario selected											
A: C2, S, F2(a), N1(b)											
1	0.53	0.16	0.13	0.14	0.00	0.01	0.02	0.47	0.21	0.08	0.04
2	0.16	0.46	0.13	0.17	0.01	0.00	0.00	0.54		0.08	
3	0.08	0.13	0.32	0.12	0.00	0.03	0.02	0.68		0.06	
4	0.08	0.11	0.14	0.33	0.01	0.01	0.02	0.67		0.06	
5	0.00	0.00	0.04	0.01	0.81	0.05	0.05	0.19	0.09	0.03	0.08
6	0.01	0.02	0.08	0.02	0.07	0.84	0.01	0.16		0.03	
7	0.01	0.01	0.02	0.08	0.07	0.00	0.83	0.17		0.03	
B: C2, S, a, b, d											
1	0.43	0.37	0.34	0.00	0.00			0.57	0.00	0.71	0.00
2	0.30	0.35	0.35	0.01	0.00			0.65		0.66	
3	0.27	0.28	0.32	0.00	0.01			0.68		0.56	
4	0.00	0.00	0.00	0.99	0.00			0.01	0.01	0.00	0.00
5	0.00	0.00	0.00	0.00	0.99			0.01		0.00	
C: C2, c, a, e, f											
1	0.36	0.40	0.43	0.00	0.00			0.64	0.00	0.82	0.00
2	0.29	0.29	0.28	0.00	0.00			0.71		0.58	
3	0.35	0.31	0.30	0.00	0.00			0.70		0.66	
4	0.00	0.00	0.00	1.00	0.00			0.00	0.00	0.00	0.00
5	0.00	0.00	0.00	0.00	1.00			0.00		0.00	
C: C2, b, a, d, k											
1	0.39	0.39	0.40	0.00	0.00			0.61	0.00	0.79	0.00
2	0.32	0.32	0.36	0.01	0.00			0.68		0.69	
3	0.28	0.28	0.24	0.00	0.00			0.76		0.57	
4	0.00	0.00	0.00	0.99	0.00			0.01	0.01	0.00	0.00
5	0.00	0.00	0.00	0.00	1.00			0.00		0.00	
C: C2, b, a, h, i											
1	0.40	0.41	0.44	0.00	0.00			0.60	0.00	0.85	0.00
2	0.27	0.28	0.28	0.01	0.00			0.72		0.56	
3	0.33	0.31	0.28	0.00	0.00			0.72		0.64	
4	0.00	0.00	0.00	0.99	0.00			0.01	0.01	0.00	0.00
5	0.00	0.00	0.00	0.00	1.00			0.00		0.00	

In the analysis of scenario set A (Fig. 2A), when Scenarios 1 through 4 were considered together, Type I and Type II error rates were 0.21 and 0.04, respectively (Table 5). The four scenarios with no recent admixture taken together did not have very high power of detection, but when there is recent admixture in the true scenario, they were very rarely selected. Type I and Type II error rates were low (≤0.01) in analyses using scenario sets B and C (Fig. 2B,C) when scenarios were grouped with respect to absence or timing of admixture. Thus, Type I and Type II error rates as assessed in DIYABC further provided support for chosen scenarios in the analyses. Also, none of the test quantities showed significant tail‐area probability (<0.05) in the posterior predictive distribution in the tests for goodness‐of‐fit of the selected scenarios after correction for multiple tests (Tables S5 and S6), indicating no significant discrepancies between the model and the data.

Posterior distributions of parameters estimated for the supported scenarios were similar to each other and consistent with the timing of the evolution of resistance to glyphosate but showed wide 95% credible intervals (Tables S7–S10). Although the prior distribution of the timing (i.e., *t*3*−tb*3, *t*4*−tb*4, and *t*5*−tb*) of the start of expansion (spread) of resistant populations was not restricted to the period after the advent of glyphosate, the estimates were well within the time period of glyphosate selection. The start of expansion (i.e., resistance spread) was estimated to be between 31 and 14 generations ago in the lineages leading to resistant populations, based on the mean of the posterior distribution. For the lineages leading to recombinant MLGs i and k, the timing of admixture with other resistant MLGs was estimated to be 32 and 31 generations ago, respectively, and the start of expansion estimated to be 10 and nine generations ago, respectively. Also, the posterior distributions of bottleneck effective population sizes (i.e., *Nb*3, *Nb*4, *Nb*5 and *Nb*) were smaller than, and did not overlap with, effective population size prior to bottleneck (*N*_*e*_) or the expansion effective population size in resistant populations (*N*_*r*_), consistent with the model. The timing of the start of bottleneck (*t*3, *t*4, *t*5*, t*2*a*, and *t*2*b*) leading to population *S* and MLGs a, b, c, d, e, and f was estimated to be around 100 years ago, which is before glyphosate use in agriculture. In contrast, in the susceptible population from the northern gene pool, C2, (i.e.*, t*1*a* and *t*1*b*), it was recent and within the time period for glyphosate selection.

## Discussion

### Selection and evolution of glyphosate resistance

The spatial structuring of phenotypic response (resistance or susceptibility) to glyphosate in the sampled populations can be explained by variation in selection pressure by glyphosate. The recent spread of glyphosate resistance in *C*. *canadensis* of California mainly happened in the southern areas of the Central Valley (Fig. 3A; Hanson et al. 2009). Our results suggest that it was associated with increased selection by glyphosate due to recent regulatory restrictions on the use of herbicides other than glyphosate in GWPA that came into effect in 2004, as hypothesized by Shrestha et al. (2007). Although the correlation between the size of GWPA in a county and the frequency of resistant plants was significant at the county level, there were several exceptions to the trend, indicating that selection at the field level also affects evolution of resistance. Similar regional and local influences were observed for acetyl‐coenzyme A carboxylase‐inhibiting herbicides in *Alopecurus myosuroides* (Délye et al. 2010).

*Conyza canadensis* populations with glyphosate‐resistant plants were detected throughout the Central Valley and surrounding areas (Fig. 3A), in agreement with Hanson et al. (2009). Glyphosate has been used for decades as the primary herbicide for weed control in orchards and vineyards, and noncrop areas of the Central Valley (CADPR 2009). Undoubtedly, resistance to glyphosate is an adaptive response to this use pattern in the Central Valley. Based on the results of the ABC analysis, there were multiple independent origins of glyphosate resistance in California populations of *C*. *canadensis*. These original resistant populations were estimated to have undergone expansion, which modeled the response to positive selection in this analysis, between 31 and 14 generations or years ago (Tables S7–S10). The results indicate that glyphosate resistance in *C*. *canadensis* of California was present many years before it was first detected in 2005 by Shrestha et al. (2007). Further, our results suggest that diversity in weed control practices, prior to the regulation of herbicides within GWPA, probably kept glyphosate‐resistant individuals at frequencies too low to be detected earlier.

### Population structure, population history, and evolution of glyphosate resistance

The distance‐based clustering of populations (Fig. 4) and pairwise *F*_ST_ analysis (Table S3) showed two groups of glyphosate‐resistant populations (Fig. 4), indicating recent resistance spread among populations within each group. The spread of resistance among populations was indicated within the counties with the greater areas designated as GWPA (Table 1). Increased selection for glyphosate resistance is expected in GWPA, and larger areas designated as GWPA increase landscape uniformity for greater selection pressure within regions. Thus, high intensity and uniformity of selection pressure for glyphosate resistance in these areas likely provided suitable habitats for the resistant plants to spread. Most individuals from highly resistant populations assigned highly to three of the gene pools at *K *=* *14 in the Bayesian clustering INSTRUCT analysis, indicating at least three independent origins of the glyphosate resistance trait. The analysis of MLGs in highly resistant populations indicated possible additional origins of resistance (Table 3). The ABC analyses also strongly supported scenarios for independent origins of resistant populations and MLGs (Table 4). Multiple origins of herbicide resistance have often been revealed by the identification of distinct mutations in the gene encoding the herbicide's target enzyme (reviewed in Powles and Yu 2010). When the molecular mechanism of herbicide resistance at the DNA sequence level is unknown, as in the case of *C. canadensis*, ABC analysis offers an approach that differentiates between single or multiple origins of resistance and allows insight into the dynamics of the evolution and spread of resistance based on neutral marker variation.

The spatial structuring of microsatellite variation and response to glyphosate (Figs 3A,B and 5B) suggests patterns created by historical processes overlain with contemporary adaptive processes (Hairston et al. 2005; Carroll et al. 2007) associated with the evolution and spread of resistance to the herbicide. Bayesian clustering identified 14 genetic clusters that were organized into two clusters at the highest hierarchical level (Fig. 5A,B). The clustering at *K* = 2 probably captured the historical level of population structuring along latitudinal gradients across the Central Valley, whereas the more fine‐level population structure at *K* = 14 captured the effects of recent selection and demographic spread of resistance. In the Bayesian clustering analysis, the multimodality we observed likely reflects a population structure with numerous selfing lineages and rare genetic exchange among them over a long period of time aided by human dispersal of seeds among agricultural fields and roadsides (e.g., St. Onge et al. 2011). Development of such complex relationships among individuals suggests a long‐term presence of the gene pools in the sampled region and that the resistance to glyphosate that became widespread originated in the southern part of the Central Valley.

There was no significant correlation between the frequency of resistant plants within populations and the effective selfing rate or within‐population genetic diversity, indicating that glyphosate‐resistant populations on the average do not differ from susceptible populations in terms of the dispersal as well as frequencies and/or severity of bottleneck events in their population histories. Genetic diversity over all populations was high, suggesting that rare outcrossing events may play a significant role in founding new populations and possibly adaptation to heterogeneous environments (Clements et al. 2004; Porcher et al. 2006) in both resistant and susceptible populations. Interestingly, a notably abundant and widespread multi‐locus genotype in each of the two groups of resistant populations suggests that the two genotypes may be more invasive (Zepeda‐Paulo et al. 2010) or older than other resistant genotypes.

Parameters estimated for the most highly supported scenario(s) across multiple ABC analyses using different populations or MLGs provided further insights into the population histories of resistant and susceptible populations. The timing of increase in *N*_*e*_ of resistant populations coincided with the timing of glyphosate use in agriculture and resulting evolution of resistance, indicating that the increase in *N*_*e*_ should be interpreted as positive selection for glyphosate resistance. On the other hand, the timing of the start of a bottleneck (major reduction in *N*_*e*_) was estimated to be around 100 years ago in lineages leading to all populations and MLGs (*t*2*a*,* t*2*b*,* t*3, *t*4, and *t*5) with the exception of the susceptible population C2. Our finding that population expansion (increase in *N*_*e*_) in resistant populations/MLGs was estimated to have occurred only after glyphosate use in agriculture, despite the bottleneck approximately 100 years ago, indicates a long period of successful management of *C. canadensis* populations until the evolution of resistance, consistent with observations of recent increases in the abundance of the species (Shrestha et al. 2007; Hanson et al. 2009; K. Hembree unpublished data; S. Wright unpublished data) and the decades of use of glyphosate in orchards, vineyards, and noncrop areas with no detected resistance. Interestingly, reduction in *N*_*e*_ of susceptible population C2 from northern Central Valley was later and estimated to have occurred since the advent of glyphosate or about 14 years prior to it, rather than 100 years ago as was the case for the other three populations. The more recent reduction in *N*_*e*_ in a susceptible population suggests strong selection by one or more weed management measures although recent colonization from a source with much larger *N*_*e*_ may also be the cause. Investigations into the history of management practices in such populations may provide insight into options for herbicide resistance management.

### Management of glyphosate resistance

Glyphosate resistance in *C. canadensis* populations was positively correlated to the size of GWPA in each county where glyphosate use, and thus selection for resistance, has increased recently. Genetic evidence of spread of resistance was found in counties with larger area designated as GWPA, consistent with greater landscape uniformity in high selection pressure promoting resistance spread. The reliance on glyphosate for weed control in GWPA, the multiple independent origins of resistance, and the wind dispersal of resistant seed long‐ and short‐distances (Dauer et al. 2007) provided an ideal condition for the rapid evolution and spread of glyphosate resistance across the region, as is evident in the observed widespread distribution of resistant populations across the southern Central Valley. However, a return to using herbicides with potentially negative environmental impacts, which once effectively controlled *C*. *canadensis*, is no longer desirable in this area, and growers are quickly running out of options (Shrestha et al. 2007). Integrated weed management approaches incorporating safer alternative herbicides and nonchemical methods and applied at a regional‐scale, such as the landscape‐level approaches of coordinating management among regional growers to reduce landscape uniformity, as discussed by Dauer et al. (2009b), are needed. Constant vigilance to keep selection pressure low by diversifying weed management practices (Powles 2008) at all locations is required to keep resistance and *C*. *canadensis* under control.

## Data archiving statement

Data deposited in the Dryad repository: doi:10.5061/dryad.s21k2.

## Supporting information

Supplemental data
