# Supplementary material for: Evolution and spread of glyphosate resistance in Conyza canadensis in California
Source: Evol Appl. 2013 Mar 11;6(5):761–77. doi: 10.1111/eva.12061 (PMC5779124; doi:10.1111/eva.12061)
Supplement: Supplementary file 1 — Supplemental data [file EVA-6-761-s001.pdf]

Supporting Information

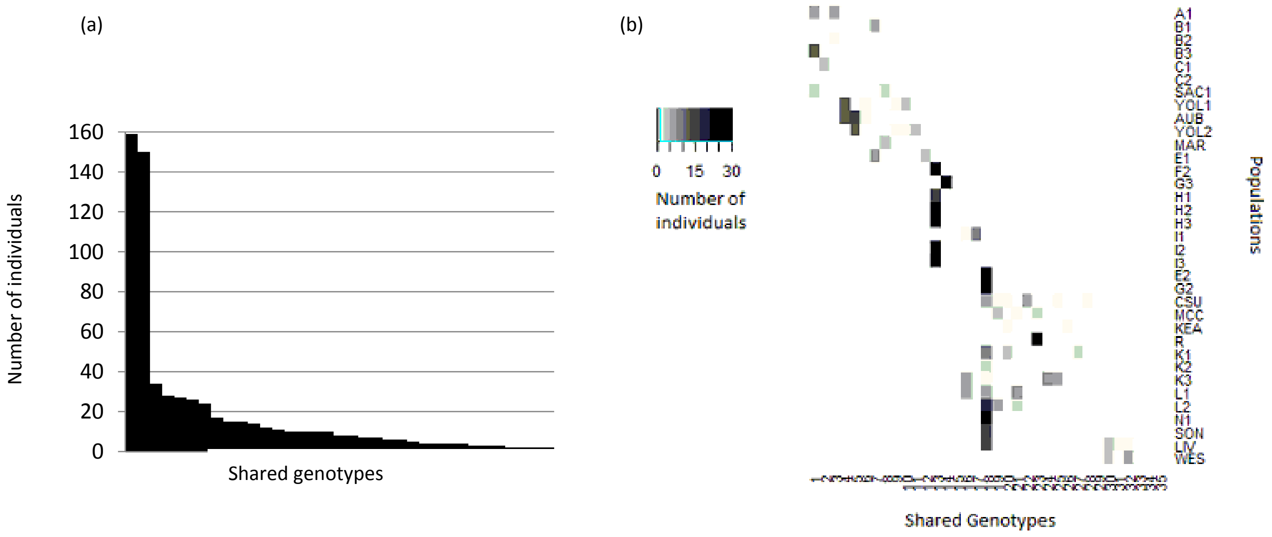

Fig. S1 (a) The number of individuals found with identical genotypes that are shared across populations in horseweed, and (b) a heatmap of the 35 shared genotypes showing their distribution in sampled populations. Genotypes 13 and 18 are the two most widespread genotypes.

Table S1 Characteristics of the six new microsatellite markers for *Conyza canadensis*.

Marker	PCR primer sequence (5' to 3')	GenBank Accession No.	Repeat motif	Amplicon size (bp)
HWSSR01	F: TATGTTGTACGACTGACTGAGATC<br>R: CCATTGACTGTAGACCAGTGTG	JX440849	$(CTAT)_{21}$	176
HWSSR03	F: TTGACTCCAACTCGTAGTGTATG<br>R: ACGTTAAATCTCTCGTGTCCTTC	JX440851	$(TG)_7(TA)_4TG(TA)_2TG(TA)_5$ $AG(TATATG)_8$	190
HWSSR04	F: GGAAAACTCCTGTCATAGTATTAGC<br>R: ATTAAAATCTAGCAAGGCCGAAC	JX440852	$(AAT)_{18}$	229
HWSSR09	F: CATGAGTTTGAGTTATCCCAGAT<br>R: CGAATACTTTCAATGCTTACGAC	JX440857	$(TTAAT)_6$	202
HWSSR11	F: ATCGTTGACATCTGACTCTGC<br>R: GATTCTTGCTCTGGTTCCTTG	JX440859	$(GAT)_{15}$	190
HWSSR12	F: CATAACAAACGGATTAGTGGCG<br>R: ATTATTGACGACCAACAACACC	JX440860	$(TAA)_{15}$	273

Table S2 Genetic diversity detected at 12 microsatellite loci in 1300 individuals of *Conyza canadensis*. Allele sizes (bp); *T*A, total number of alleles detected; H_O, observed heterozygosity; H_S, expected heterozygosity within populations; H_T, expected heterozygosity over all samples. F_{IS}, Wright's inbreeding coefficient, and F_{ST}, Wright's fixation index (Weir & Cockerham 1984), were averaged over 44 populations from California.

Locus	Allele sizes (bp)	*TA*	H_O	H_S	H_T	F_{IS}	F_{ST}
HW02	158-170	7	0.009	0.081	0.096	0.882***	0.163***
HW06	209-219	7	0.012	0.125	0.269	0.902***	0.542***
HW07	206-228	5	0.005	0.158	0.383	0.966***	0.593***
HW14	191-204	7	0.008	0.047	0.053	0.850***	0.127***
HW17	144-180	17	0.014	0.364	0.850	0.962***	0.576***
HW29	148-185	2	0.000	0.003	0.003	1.000**	0.034*
HWSSR01	121-228	21	0.016	0.323	0.774	0.950***	0.588***
HWSSR03	155-213	9	0.011	0.230	0.648	0.953***	0.650***
HWSSR04	217-286	23	0.024	0.445	0.894	0.948***	0.510***
HWSSR09	189-205	3	0.001	0.021	0.081	0.962***	0.753***
HWSSR11	172-258	10	0.015	0.322	0.642	0.955***	0.507***
HWSSR12	167-288	14	0.019	0.393	0.86	0.951***	0.550***
Mean		10.4	0.011	0.209	0.463	0.947	0.554

P values: ****P* < 0.001, ***P* < 0.01, **P* < 0.05.

Table S3 Pairwise FST values between Conyza canadensis. Negative F_{ST} estimates obtained (-0.02 for H2 vs. H3, H2 vs. I3, and H3 vs. I3) are represented as zero and were all non-significant.

	A2	AUB	B1	B2	B3	R	S	BOD1	BOD2	C1	C2	CRU	CSU	E1	E2	F2	G2	G3	H1	H2	H3	I1	I2	I3	K1	K2	K3	KEA	L1	L2	LIV	MAR	MCC	MEN1	MEN2	MON2	N1	NAP	SAC	SON	WES	YOL1	YOL2
A1	*	*	*	*	*	*	*	*	*	*	*	*	*	*	*	*	*	*	*	*	*	*	*	*	*	*	*	*	*	*	*	*	*	*	*	*	*	*	*	*	*	*	*
A2	0.00	*	*	*	*	*	*	*	*	*	*	*	*	*	*	*	*	*	*	*	*	*	*	*	*	*	*	*	*	*	*	*	*	*	*	*	*	*	*	*	*	*	*
AUB	0.43	0.00	*	*	*	*	*	*	*	*	*	*	*	*	*	*	*	*	*	*	*	*	*	*	*	*	*	*	*	*	*	*	*	*	*	*	*	*	*	*	*	*	*
B1	0.21	0.33	0.00	*	*	*	*	*	*	*	*	*	*	*	*	*	*	*	*	*	*	*	*	*	*	*	*	*	*	*	*	*	*	*	*	*	*	*	*	*	*	*	*
B2	0.30	0.40	0.25	0.00	*	*	*	*	*	*	*	*	*	*	*	*	*	*	*	*	*	*	*	*	*	*	*	*	*	*	*	*	*	*	*	*	*	*	*	*	*	*	*
B3	0.33	0.54	0.21	0.38	0.00	*	*	*	*	*	*	*	*	*	*	*	*	*	*	*	*	*	*	*	*	*	*	*	*	*	*	*	*	*	*	*	*	*	*	*	*	*	*
R	0.68	0.87	0.55	0.79	0.77	0.00	*	*	*	*	*	*	*	*	*	*	*	*	*	*	*	*	*	*	*	*	*	*	*	*	*	*	*	*	*	*	*	*	*	*	*	*	*
S	0.66	0.88	0.54	0.79	0.70	1.00	0.00	*	*	*	*	*	*	*	*	*	*	*	*	*	*	*	*	*	*	*	*	*	*	*	*	*	*	*	*	*	*	*	*	*	*	*	*
BOD1	0.70	0.89	0.63	0.79	0.79	1.00	1.00	0.00	*	*	*	*	*	*	*	*	*	*	*	*	*	*	*	*	*	*	*	*	*	*	*	*	*	*	*	*	*	*	*	*	*	*	*
BOD2	0.44	0.62	0.36	0.52	0.50	0.73	0.71	0.68	0.00	*	*	*	*	*	*	*	*	*	*	*	*	*	*	*	*	*	*	*	*	*	*	*	*	*	*	*	*	*	*	*	*	*	*
C1	0.33	0.42	0.19	0.34	0.37	0.80	0.82	0.82	0.54	0.00	*	*	*	*	*	*	*	*	*	*	*	*	*	*	*	*	*	*	*	*	*	*	*	*	*	*	*	*	*	*	*	*	*
C2	0.27	0.35	0.15	0.25	0.27	0.70	0.74	0.74	0.48	0.19	0.00	*	*	*	*	*	*	*	*	*	*	*	*	*	*	*	*	*	*	*	*	*	*	*	*	*	*	*	*	*	*	*	*
CRU	0.47	0.67	0.41	0.58	0.57	0.78	0.71	0.77	0.51	0.59	0.54	0.00	*	*	*	*	*	*	*	*	*	*	*	*	*	*	*	*	*	*	*	*	*	*	*	*	*	*	*	*	*	*	*
CSU	0.42	0.59	0.28	0.52	0.44	0.50	0.64	0.74	0.52	0.50	0.44	0.51	0.00	*	*	*	*	*	*	*	*	NS	*	*	NS	*	NS	*	NS	NS	*	*	NS	*	*	*	*	*	*	*	*	*	*
E1	0.36	0.57	0.19	0.47	0.42	0.68	0.68	0.75	0.43	0.44	0.38	0.53	0.42	0.00	*	*	*	*	*	*	*	*	*	*	*	*	*	*	*	*	*	*	*	*	*	*	*	*	*	*	*	*	*
E2	0.69	0.87	0.62	0.81	0.75	0.96	0.96	0.97	0.75	0.81	0.74	0.75	0.41	0.73	0.00	*	NS	*	*	*	*	NS	*	*	*	*	*	*	*	NS	*	*	*	*	*	*	NS	*	*	*	*	*	*
F2	0.59	0.86	0.56	0.74	0.69	1.00	1.00	1.00	0.70	0.77	0.66	0.75	0.70	0.66	0.96	0.00	*	*	*	NS	NS	*	NS	NS	*	*	*	*	*	*	*	*	*	*	*	*	*	*	*	*	*	*	*
G2	0.58	0.77	0.51	0.70	0.63	0.86	0.86	0.89	0.66	0.70	0.64	0.66	0.28	0.63	0.03	0.87	0.00	*	*	*	*	*	*	*	*	*	*	*	*	NS	*	*	*	*	*	*	NS	*	*	NS	*	*	*
G3	0.51	0.70	0.50	0.60	0.62	0.89	0.87	0.89	0.64	0.66	0.58	0.60	0.59	0.61	0.83	0.84	0.72	0.00	*	*	*	*	*	*	*	*	*	*	*	*	*	*	*	*	*	*	*	*	*	*	*	*	*
H1	0.29	0.49	0.24	0.40	0.30	0.59	0.60	0.69	0.44	0.39	0.32	0.43	0.28	0.32	0.59	0.31	0.48	0.45	0.00	NS	NS	*	NS	NS	*	*	*	*	*	*	*	*	*	*	*	*	*	*	*	*	*	*	*
H2	0.52	0.78	0.49	0.66	0.60	0.93	0.92	0.94	0.65	0.68	0.58	0.69	0.64	0.59	0.90	0.01	0.81	0.77	0.22	0.00	NS	*	NS	NS	*	*	*	*	*	*	*	*	*	*	*	*	*	*	*	*	*	*	*
H3	0.52	0.78	0.49	0.66	0.60	0.93	0.92	0.94	0.65	0.68	0.58	0.69	0.64	0.59	0.90	0.01	0.80	0.77	0.23	0.00	0.00	*	NS	NS	*	*	*	*	*	*	*	*	*	*	*	*	*	*	*	*	*	*	*
I1	0.37	0.54	0.27	0.46	0.38	0.57	0.59	0.68	0.45	0.45	0.41	0.45	0.10	0.39	0.35	0.62	0.24	0.48	0.26	0.56	0.56	0.00	*	*	NS	*	NS	*	*	*	*	*	*	*	*	*	*	*	*	*	*	*	*
I2	0.41	0.65	0.38	0.53	0.48	0.83	0.81	0.84	0.56	0.55	0.46	0.59	0.54	0.47	0.81	0.09	0.71	0.65	0.13	0.03	0.03	0.47	0.00	NS	*	*	*	*	*	*	*	*	*	*	*	*	*	*	*	*	*	*	*
I3	0.51	0.77	0.48	0.65	0.59	0.92	0.91	0.93	0.64	0.67	0.57	0.68	0.63	0.58	0.90	0.02	0.80	0.76	0.21	0.00	0.00	0.55	0.02	0.00	*	*	*	*	*	*	*	*	*	*	*	*	*	*	*	*	*	*	*
K1	0.40	0.57	0.29	0.50	0.42	0.52	0.64	0.72	0.51	0.48	0.43	0.50	0.00	0.43	0.35	0.69	0.23	0.57	0.27	0.62	0.62	0.09	0.53	0.62	0.00	*	NS	*	NS	NS	*	*	NS	*	*	*	*	*	*	*	*	*	*
K2	0.28	0.45	0.16	0.38	0.33	0.41	0.41	0.62	0.39	0.37	0.32	0.37	0.17	0.27	0.54	0.57	0.44	0.48	0.21	0.51	0.51	0.19	0.41	0.50	0.19	0.00	*	*	*	*	*	*	*	*	*	*	*	*	*	*	*	*	*
K3	0.37	0.55	0.24	0.47	0.39	0.40	0.59	0.71	0.48	0.45	0.40	0.49	0.08	0.38	0.58	0.67	0.46	0.57	0.25	0.60	0.60	0.16	0.50	0.59	0.08	0.11	0.00	NS	NS	*	*	*	NS	*	*	*	*	*	*	*	*	*	*
KEA	0.41	0.60	0.29	0.52	0.47	0.56	0.59	0.74	0.50	0.51	0.45	0.47	0.17	0.41	0.62	0.70	0.50	0.56	0.29	0.64	0.64	0.23	0.53	0.62	0.20	0.17	0.17	0.00	*	*	*	*	*	*	*	*	*	*	*	*	*	*	*
L1	0.45	0.61	0.32	0.54	0.47	0.53	0.69	0.76	0.55	0.53	0.46	0.55	0.07	0.47	0.55	0.74	0.42	0.63	0.32	0.68	0.68	0.20	0.59	0.67	0.03	0.22	0.09	0.21	0.00	NS	*	*	NS	*	*	*	*	*	*	*	*	*	*
L2	0.52	0.68	0.41	0.62	0.53	0.70	0.74	0.81	0.60	0.60	0.54	0.58	0.11	0.54	0.26	0.79	0.15	0.63	0.37	0.73	0.72	0.18	0.64	0.72	0.06	0.31	0.29	0.31	0.17	0.00	*	*	*	*	*	*	NS	*	*	*	*	*	*
LIV	0.36	0.55	0.28	0.46	0.39	0.67	0.64	0.71	0.45	0.43	0.39	0.45	0.24	0.37	0.38	0.63	0.25	0.47	0.27	0.57	0.56	0.16	0.47	0.56	0.21	0.27	0.30	0.35	0.33	0.25	0.00	*	*	*	*	*	NS	*	*	*	*	*	*
MAR	0.50	0.74	0.44	0.63	0.59	0.88	0.86	0.90	0.59	0.65	0.58	0.62	0.60	0.48	0.88	0.85	0.78	0.69	0.49	0.77	0.77	0.51	0.64	0.76	0.60	0.41	0.56	0.58	0.65	0.70	0.46	0.00	*	*	*	*	*	*	*	*	*	*	*
MCC	0.41	0.58	0.28	0.51	0.43	0.45	0.63	0.73	0.52	0.49	0.43	0.51	0.03	0.41	0.54	0.70	0.41	0.60	0.26	0.63	0.63	0.18	0.54	0.62	0.04	0.15	0.04	0.15	0.01	0.18	0.31	0.59	0.00	*	*	*	*	*	*	*	*	*	*
MEN1	0.63	0.84	0.46	0.70	0.68	0.98	0.98	0.98	0.60	0.73	0.66	0.75	0.69	0.59	0.96	0.98	0.88	0.86	0.61	0.90	0.90	0.60	0.78	0.89	0.69	0.53	0.65	0.68	0.73	0.78	0.64	0.82	0.69	0.00	*	*	*	*	*	*	*	*	*
MEN2	0.41	0.58	0.33	0.46	0.48	0.77	0.78	0.74	0.46	0.46	0.39	0.53	0.54	0.46	0.79	0.74	0.70	0.66	0.44	0.67	0.67	0.44	0.57	0.66	0.53	0.41	0.50	0.54	0.56	0.62	0.47	0.58	0.53	0.65	0.00	*	*	*	*	*	*	*	*
MON2	0.51	0.73	0.45	0.63	0.62	0.84	0.79	0.81	0.50	0.65	0.59	0.33	0.57	0.58	0.84	0.82	0.74	0.73	0.50	0.76	0.76	0.51	0.66	0.75	0.56	0.41	0.53	0.56	0.61	0.67	0.53	0.72	0.57	0.81	0.55	0.00	*	*	*	*	*	*	*
N1	0.73	0.91	0.66	0.84	0.80	1.00	1.00	1.00	0.78	0.86	0.79	0.79	0.46	0.77	0.00	1.00	0.08	0.87	0.64	0.94	0.94	0.40	0.86	0.93	0.41	0.59	0.63	0.67	0.60	0.32	0.44	0.91	0.59	0.99	0.83	0.87	0.00	*	*	*	*	*	*
NAP	0.59	0.77	0.53	0.67	0.67	0.92	0.93	0.93	0.70	0.65	0.60	0.71	0.59	0.66	0.86	0.91	0.75	0.76	0.57	0.85	0.85	0.52	0.75	0.84	0.57	0.53	0.62	0.62	0.64	0.63	0.56	0.80	0.61	0.88	0.70	0.80	0.90	0.00	*	*	*	*	*
SAC	0.29	0.47	0.20	0.18	0.26	0.76	0.74	0.77	0.47	0.30	0.22	0.52	0.48	0.39	0.78	0.69	0.67	0.54	0.33	0.61	0.61	0.42	0.47	0.60	0.47	0.33	0.41	0.46	0.51	0.58	0.36	0.47	0.47	0.65	0.42	0.60	0.82	0.65	0.00	*	*	*	*
SON	0.31	0.47	0.22	0.40	0.32	0.58	0.59	0.65	0.41	0.39	0.33	0.43	0.14	0.36	0.26	0.59	0.14	0.47	0.23	0.53	0.52	0.12	0.44	0.52	0.11	0.22	0.24	0.29	0.23	0.14	0.11	0.53	0.22	0.61	0.44	0.50	0.31	0.48	0.37	0.00	*	*	*
WES	0.29	0.49	0.20	0.34	0.33	0.67	0.65	0.67	0.28	0.33	0.26	0.46	0.43	0.27	0.69	0.58	0.59	0.53	0.30	0.52	0.51	0.37	0.41	0.51	0.42	0.30	0.38	0.43	0.46	0.52	0.27	0.41	0.43	0.55	0.35	0.49	0.73	0.61	0.22	0.29	0.00	*	*
YOL1	0.29	0.22	0.15	0.25	0.30	0.73	0.73	0.75	0.49	0.21	0.17	0.54	0.46	0.38	0.76	0.69	0.65	0.57	0.34	0.61	0.61	0.42	0.49	0.60	0.44	0.33	0.41	0.47	0.48	0.56	0.40	0.58	0.45	0.67	0.41	0.59	0.80	0.63	0.24	0.35	0.31	0.00	*
YOL2	0.33	0.24	0.21	0.30	0.41	0.75	0.78	0.78	0.54	0.25	0.17	0.59	0.49	0.41	0.78	0.74	0.68	0.62	0.40	0.66	0.66	0.46	0.55	0.65	0.47	0.36	0.45	0.50	0.51	0.58	0.44	0.63	0.47	0.72	0.45	0.64	0.82	0.65	0.32	0.38	0.36	0.15	0.00

Table S4 Multilocus microsatellite marker genotype data in resistant populations E2, F2, H2, H3, I1, K1, L1, L2, and N1. Multilocus genotypes that occurred more than once are identified by lowercase letters and singletons are identified by the population in which they occurred.

ID	n	HW07		HW17		HW06		HWSSR01		HW14		HWSSR12		HW02		HW29		HWSSR09		HWSSR03		HWSSR04		HWSSR11	
a	85	226	226	149	149	213	213	121	121	200	200	258	258	162	162	148	148	205	205	155	155	262	262	191	191
b	93	228	228	168	168	213	213	179	179	200	200	270	270	162	162	148	148	205	205	191	191	229	229	194	194
c	14	228	228	168	168	211	211	179	179	200	200	270	270	162	162	148	148	205	205	191	191	229	229	194	194
d	11	226	226	166	166	213	213	125	125	200	200	270	270	162	162	148	148	205	205	195	195	238	238	200	200
e	10	226	226	166	166	213	213	171	171	200	200	249	249	162	162	148	148	205	205	195	195	265	265	191	191
f	10	226	226	168	168	213	213	125	125	200	200	249	249	162	162	148	148	205	205	195	195	238	238	200	200
g	8	228	228	166	166	213	213	125	125	200	200	270	270	162	162	148	148	205	205	195	195	238	238	200	200
h	5	226	226	166	166	213	213	125	125	200	200	249	249	162	162	148	148	205	205	195	195	238	238	200	200
i	3	226	226	168	168	213	213	179	179	200	200	270	270	162	162	148	148	205	205	195	195	238	238	200	200
j	2	226	226	162	162	213	213	228	228	196	196	264	264	162	162	148	148	205	205	155	155	265	265	185	185
k	2	228	228	166	166	213	213	179	179	200	200	270	270	162	162	148	148	205	205	195	195	238	238	200	200
l	2	228	228	166	166	213	213	179	179	200	200	270	270	162	162	148	148	205	205	195	195	229	229	194	194
m	2	228	228	166	166	213	213	179	179	200	200	273	273	162	162	148	148	205	205	155	155	220	220	200	200
E2	1	226	226	155	155	213	213	121	121	200	200	258	258	162	162	148	148	205	205	155	155	262	262	191	191
F2	1	226	226	149	149	213	213	121	121	200	200	255	258	162	162	148	148	205	205	155	155	262	262	191	191
H2	1	226	226	168	168	213	213	179	179	200	200	167	167	162	162	148	148	205	205	155	155	226	226	194	194
H2	1	226	226	149	149	213	213	121	121	200	200	258	258	162	162	148	148	205	205	155	155	238	238	191	191
H2	1	227	227	166	166	213	213	179	179	200	200	270	270	162	162	148	148	205	205	184	184	262	262	200	200
H3	1	226	226	164	164	213	213	171	171	200	200	264	264	162	162	148	148	205	205	155	155	265	265	194	194
I1	1	228	228	168	168	211	211	179	183	200	200	270	270	162	162	148	148	205	205	191	191	229	229	194	194
K1	1	226	226	166	166	213	213	179	179	200	200	249	249	162	162	148	148	205	205	191	191	229	229	194	194
K1	1	226	226	166	166	213	213	171	171	200	200	270	270	162	162	148	148	205	205	195	195	265	265	191	191
K1	1	228	228	168	168	213	213	125	125	200	200	249	249	162	162	148	148	205	205	191	191	238	238	200	200
K1	1	226	226	168	168	213	213	129	129	200	200	249	249	164	164	148	148	205	205	155	155	265	265	194	194
K1	1	226	226	160	160	213	213	171	171	200	200	270	270	162	162	148	148	205	205	195	195	265	265	191	191
K1	1	226	226	168	168	213	213	125	125	200	200	270	270	162	162	148	148	205	205	191	191	238	238	200	200
K1	1	226	226	166	166	213	213	125	125	200	200	249	249	162	162	148	148	205	205	191	191	229	229	194	194
K1	1	228	228	160	160	213	213	179	179	200	200	270	270	162	162	148	148	205	205	155	191	229	229	194	194
K1	1	226	226	166	168	213	213	179	179	200	200	249	270	162	162	148	148	205	205	191	195	229	238	194	200
L1	1	226	226	168	168	213	213	179	179	200	200	270	270	162	162	148	148	205	205	191	191	238	238	194	194
L1	1	226	228	166	168	213	213	179	179	200	200	270	270	162	162	148	148	205	205	191	195	229	229	194	194
L1	1	226	226	168	168	213	213	125	166	200	200	249	267	162	162	148	148	205	205	195	195	238	238	200	200
L1	1	226	226	168	168	213	213	125	158	200	200	249	270	162	162	148	148	205	205	195	195	238	238	200	200
L1	1	228	228	166	166	213	213	125	179	200	200	270	270	162	162	148	148	205	205	195	195	238	238	200	200
L2	1	228	228	166	166	213	213	125	125	200	200	267	270	162	162	148	148	205	205	195	195	238	238	200	200

Table S5 Model checking for the four scenarios with no recent admixture (Fig. 2A). HET, mean expected heterozygosity in population; VAR, mean allelic size variance in population; DAS, shared allele distance between populations. Populations 1 through 4 correspond to P1, P2, P3, and P4. Significant tail-area probabilities for each test quantity after applying the false discovery rate correction method of Benjamini and Hochberg (1995) for multiple tests.

		probability ($t_{simulated} < t_{observed}$)							
Test quantity (t)	observed value	Scenario 1		Scenario 2		Scenario 3		Scenario 4	
HET_P1	0.2484	0.5150	ns	0.5950	ns	0.5050	ns	0.4800	ns
HET_P2	0	0.0200	ns	0.0150	ns	0.0100	ns	0.0050	ns
HET_P3	0.0028	0.3600	ns	0.3400	ns	0.3800	ns	0.2200	ns
HET_P4	0	0.1450	ns	0.1700	ns	0.1650	ns	0.1750	ns
VAR_P1	3.2121	0.7900	ns	0.8400	ns	0.7900	ns	0.7100	ns
VAR_P2	0	0.0200	ns	0.0150	ns	0.0100	ns	0.0050	ns
VAR_P3	0.0014	0.3400	ns	0.3200	ns	0.3500	ns	0.2100	ns
VAR_P4	0	0.1550	ns	0.1700	ns	0.1700	ns	0.1750	ns
DAS_P1&2	0.5287	0.6250	ns	0.7100	ns	0.6550	ns	0.6200	ns
DAS_P1&3	0.6322	0.6900	ns	0.7700	ns	0.7800	ns	0.7200	ns
DAS_P1&4	0.4195	0.3800	ns	0.3000	ns	0.3350	ns	0.4200	ns
DAS_P2&3	0.5833	0.7350	ns	0.6600	ns	0.7350	ns	0.6950	ns
DAS_P2&4	0.5	0.4350	ns	0.4550	ns	0.3000	ns	0.3700	ns
DAS_P3&4	0.4167	0.3800	ns	0.3500	ns	0.2600	ns	0.2700	ns

Table S6 Model checking for the best scenario(s) in DIYABC analyses (Cornuet et al. 2008, 2010) with scenario sets B and C (Figs 2B and 2C) in *Conyza canadensis*. HET, mean expected heterozygosity in population; VAR, mean allelic size variance in population; DAS, shared allele distance between populations. Populations 1 through 5 correspond to P1, P2, P3, P4 and P5. Significant tail-area probabilities ($t_{simulated} < t_{observed}$) for each test quantity after applying the false discovery rate correction method of Benjamini and Hochberg (1995) for multiple tests.

	C2,S,a,b,d					C2,c,a,e,f		C2,b,a,d,k		C2,b,a,h,i	
		Scenario 1		Scenario 2		Scenario 2		Scenario 4		Scenario 4	
Test quantity (t)	observed value	probability		probability		observed value	probability	observed value	probability	observed value	probability
HET_P1	0.2484	0.615	ns	0.545	ns	0.2484	0.440	0.2484	0.340	0.2484	0.330
HET_P2	0	0.005	ns	0.030	ns	0	0.235	0	0.230	0	0.130
HET_P3	0	0.170	ns	0.145	ns	0	0.110	0	0.205	0	0.185
HET_P4	0	0.135	ns	0.095	ns	0	0.165	0	0.260	0	0.270
HET_P5	0	0.140	ns	0.175	ns	0	0.220	0	0.260	0	0.270
VAR_P1	3.2121	0.830	ns	0.760	ns	3.2121	0.640	3.2121	0.730	3.2121	0.630
VAR_P2	0	0.005	ns	0.030	ns	0	0.235	0	0.235	0	0.130
VAR_P3	0	0.170	ns	0.145	ns	0	0.110	0	0.210	0	0.195
VAR_P4	0	0.145	ns	0.095	ns	0	0.170	0	0.260	0	0.270
VAR_P5	0	0.140	ns	0.175	ns	0	0.220	0	0.260	0	0.270
DAS_P1&2	0.5287	0.720	ns	0.680	ns	0.3391	0.270	0.4195	0.510	0.4195	0.480
DAS_P1&3	0.6322	0.760	ns	0.560	ns	0.6322	0.730	0.6322	0.690	0.6322	0.805
DAS_P1&4	0.4195	0.395	ns	0.460	ns	0.523	0.790	0.5833	0.800	0.5776	0.880
DAS_P1&5	0.5833	0.800	ns	0.845	ns	0.5776	0.865	0.4195	0.460	0.5029	0.750
DAS_P2&3	0.5833	0.745	ns	0.740	ns	0.3333	0.270	0.4167	0.375	0.4167	0.530
DAS_P2&4	0.5	0.350	ns	0.435	ns	0.3333	0.185	0.5	0.325	0.4167	0.330
DAS_P2&5	0.6667	0.670	ns	0.710	ns	0.4167	0.270	0.6667	0.375	0.6667	0.365
DAS_P3&4	0.4167	0.340	ns	0.385	ns	0.5833	0.825	0.5	0.605	0.5	0.735
DAS_P3&5	0.5	0.555	ns	0.520	ns	0.5	0.645	0.4167	0.425	0.5	0.740
DAS_P4&5	0.5	0.425	ns	0.395	ns	0.6667	0.640	0.8333	0.445	0.75	0.445

Table S7 parameter estimation Prior and posterior parameter values in analysis of scenario set A (Fig. 2A) for Scenario 1 and Scenario 1 through 4 analyzed together in DIYABC (Cornuet et al. 2008, 2010). CI, credible interval; population size estimates (Nr, Nb3, Nb4, Nb, and Ne) refer to the effective breeding population; μ, mean mutation rate; time in generations (t3, t4, b3, b4, t5, t6, t1a, t1b, t2a, t2b, t7, and t8); *P*, mean coefficient of the geometric distribution under the Generalized Stepwise Mutation (GSM model); μsni, mean single nucleotide insertion-deletion mutation rate.

			Scenario 1					Scenario 1-4				
	prior values		mean	median	mode	95% CI		mean	median	mode	95% CI	
Resistant populations												
t3	1	150	105	112	143	31	149	107	114	148	32	149
t4	1	150	103	108	149	29	149	104	110	148	31	149
b3	1	150	74	76	104	8	136	76	78	89	8	136
b4	1	150	89	94	118	14	141	89	94	112	13	141
t5	150	10000	4930	4920	3950	716	9030	4740	4680	3940	761	8920
t6	150	10000	5900	6140	6720	1300	9310	5840	6030	6540	1350	9320
Nr	10	10^5	53300	54900	91300	2730	97900	49700	49600	10	2060	97600
Nb3	2	100	22	14	3	2	86	23	14	3	2	86
Nb4	2	100	24	15	2	2	89	22	13	2	2	85
Nb	2	100	66	71	95	10	99	64	68	96	9	99
Susceptible populations												
t1a	2	150	37	23	2	2	132	39	25	3	2	133
t1b	2	150	35	23	3	2	129	38	25	2	2	132
t2a	2	150	101	110	148	14	149	101	111	148	14	149
t2b	2	150	96	105	145	11	149	100	109	146	13	149
r1	0.001	0.999	0.639	0.674	0.958	0.117	0.979	0.593	0.620	0.936	0.085	0.971
r2	0.001	0.999	0.517	0.511	0.996	0.008	0.994	0.494	0.485	0.001	0.007	0.993
t7	150	10000	5770	6010	6770	923	9360	5570	5790	6800	796	9320
t8	150	10000	1260	779	160	171	5540	1410	885	157	176	5840
Resistant and susceptible populations												
ta	150	10000	8720	9020	9960	5930	9960	8790	9080	9890	6050	9960
Ne	10	10000	2030	1310	383	120	8130	2230	1390	439	122	8770
μ	$1E^{-5}$	$1E^{-4}$	$4.7E^{-05}$	$4.5E^{-05}$	$3.6E^{-05}$	$1.7E^{-05}$	$9.2E^{-05}$	$4.6E^{-05}$	$4.3E^{-05}$	$3.3E^{-05}$	$1.6E^{-05}$	$9.2E^{-05}$
P	$1E^{-1}$	$7E^{-1}$	$6.3E^{-01}$	$6.7E^{-01}$	$7.0E^{-01}$	$2.1E^{-01}$	$7.0E^{-01}$	$6.1E^{-01}$	$6.5E^{-01}$	$7.0E^{-01}$	$1.0E^{-01}$	$7.0E^{-01}$
μsni	$1E^{-9}$	$7E^{-5}$	$1.4E^{-06}$	$1.7E^{-07}$	$1.0E^{-09}$	$1.0E^{-09}$	$8.5E^{-06}$	$1.5E^{-06}$	$2.0E^{-07}$	$2.0E^{-09}$	$1.0E^{-09}$	$8.6E^{-06}$

Table S8 Prior and posterior parameter values obtained in DIYABC (Cornuet et al. 2008, 2010) in *Conyza canadensis* for Scenario 1 and Scenario 2 of scenario set B (Fig. 2B) tested using populations/multilocus genotypes, C2, S, a, b, and d, as P1 through P5, respectively. CI, credible interval; population size estimates (Nb3, Nb4, Nb5, Nb, Nr3, Nr4, Nr5 and Ne) refer to the effective breeding population; µ, mean mutation rate; time in generations (t3, t4, t5, b3, b4, b5, t6, t1a, t1b, t2a, t2b, t7, t9, t10, t10h, and t11); *P*, mean coefficient of the geometric distribution under the Generalized Stepwise Mutation (GSM model); µsni, mean single nucleotide insertion-deletion mutation rate.

			Scenario 1					Scenario 2				
	prior values		mean	median	mode	95% CI		mean	median	mode	95% CI	
Resistant populations												
t3	1	150	99	104	146	26	148	109	116	150	37	149
t4	1	150	99	104	150	26	149	101	106	147	27	149
t5	1	150	97	102	122	23	148	103	109	148	27	149
b3	1	150	83	87	115	10	139	93	99	123	14	143
b4	1	150	83	87	109	11	140	85	90	103	11	141
b5	1	150	73	75	91	7	136	82	85	108	8	140
t10	150	10000	5210	5220	5240	1020	9020	-	-	-	-	-
t10h	150	10000	-	-	-	-	-	4300	4240	4360	715	8150
t6	150	10000	5980	6160	6750	1470	9350	7120	7340	7750	3580	9540
t7	150	10000	6830	7270	8480	1590	9630	5930	6180	7270	1110	9440
Nb3	2	100	27	18	3	2	90	27	18	3	2	91
Nb4	2	100	27	19	5	2	90	27	18	3	2	90
Nb5	2	100	30	21	3	2	92	28	19	3	2	90
Nr3	10	$1E^6$	49400	49300	1010	2060	97000	50400	50700	82200	2470	97300
Nr4	10	$1E^6$	48200	47300	5050	2220	97400	46300	45000	3000	1780	96800
Nr5	10	$1E^6$	50700	50900	73800	2580	97500	47100	45800	12600	2180	97300
r5	0.001	0.009	-	-	-	-	-	0.552	0.575	0.710	0.041	0.976
Susceptible populations												
t1a	2	150	37	24	2	2	132	36	23	3	2	130
t1b	2	150	43	30	3	3	137	37	25	3	2	132
t2a	2	150	93	100	140	10	148	96	104	142	11	149
t2b	2	150	94	100	142	11	148	98	107	147	12	149
r1	0.001	0.009	0.662	0.706	0.845	0.134	0.976	0.613	0.640	0.650	0.108	0.972
r2	0.001	0.009	0.452	0.380	0.003	0.006	0.990	0.484	0.459	0.002	0.008	0.991
t9	150	10000	5560	5700	5720	1050	9250	4730	4700	4700	756	8910
t11	150	10000	1300	821	153	171	5450	1260	808	197	176	5120

Nb	2	100	75	82	98	19	100	73	80	99	16	100
Resistant and susceptible populations												
ta	150	10000	9030	9270	9830	6800	9970	9010	9260	9950	6680	9970
Ne	10	10000	1060	630	220	78	5300	1330	828	354	92	6180
μ	1E^{-05}	1E^{-04}	5.1E^{-05}	4.9E^{-05}	3.6E^{-05}	1.9E^{-05}	9.3E^{-05}	5.0E^{-05}	4.8E^{-05}	3.6E^{-05}	1.9E^{-05}	9.3E^{-05}
P	1E^{-01}	1E^{-07}	5.7E^{-01}	6.3E^{-01}	7.0E^{-01}	1.0E^{-01}	7.0E^{-01}	5.7E^{-01}	6.2E^{-01}	7.0E^{-01}	1.0E^{-01}	7.0E^{-01}
μsni	1E^{-09}	1E^{-05}	1.3E^{-06}	1.7E^{-07}	1.0E^{-09}	1.0E^{-09}	8.3E^{-06}	1.3E^{-06}	1.6E^{-07}	1.0E^{-09}	1.0E^{-09}	8.3E^{-06}

Table S9 Prior and posterior parameter values obtained in DIYABC (Cornuet et al. 2008, 2010) in *Conyza canadensis* for Scenario 2 of scenario set C (Fig. 2C) tested using populations/multilocus genotypes, C2,c,a,e, and f, as P1 through P5, respectively. CI, credible interval; population size estimates (Nb2, Nb3, Nb4, Nb5, Nb, Nr, Nr4, Nr5 and Ne) refer to the effective breeding population; µ, mean mutation rate; time in generations (t2, t3, t4, t5, th5, b3, b4, b5, t1a, t1b, t6, t7, and t9); *P*, mean coefficient of the geometric distribution under the Generalized Stepwise Mutation (GSM model); µsni, mean single nucleotide insertion-deletion mutation rate.

			Scenario 2 (C2,c,a,e,f)				
	prior values		mean	median	mode	95% CI	
Resistant populations							
t2	1	150	113	120	143	41	149
t3	1	150	106	112	147	32	149
t4	1	150	108	115	148	35	149
t5	1	150	106	113	149	32	149
b2	1	150	91	97	117	14	142
b3	1	150	81	85	85	10	138
b4	1	150	79	81	97	10	139
b5	1	150	83	87	109	10	141
th5	150	10000	2670	2360	1760	419	6520
r5	0.001	0.009	0.398	0.357	0.199	0.019	0.943
t6	150	10000	6800	6950	7050	3360	9420
t9	150	10000	4890	4880	4140	798	8940
Nb2	2	100	18	10	2	2	78
Nb3	2	100	19	11	2	2	80
Nb4	2	100	19	11	2	2	80
Nb5	2	100	18	10	2	2	79
Nr	10	$1E^{+06}$	46600	44700	12000	1940	96600
Nr4	10	$1E^{+06}$	45200	43200	3260	1920	96600
Nr5	10	$1E^{+06}$	46000	43800	1120	1870	96800
Susceptible populations							
t1a	2	150	54	44	5	3	142
t1b	2	150	54	44	7	3	141
t7	150	10000	4520	4390	3180	588	8940
r1	0.001	0.009	0.583	0.600	0.666	0.091	0.962
Nb	2	100	79	86	99	24	100
Resistant and susceptible populations							
ta	150	10000	8900	9160	9970	6530	9970
Ne	10	10000	2030	1460	687	183	7460
µ	$1E\text{-}^{05}$	$1E^{-04}$	$5.4E^{-05}$	$5.2E^{-05}$	$4.2E^{-05}$	$2.1E^{-05}$	$9.6E^{-05}$
P	$1E^{-01}$	$1E^{-07}$	$6.3E^{-01}$	$6.7E^{-01}$	$7.0E^{-01}$	$2.5E^{-01}$	$7.0E^{-01}$
µsni	$1E^{-09}$	$1E^{-05}$	$1.5E^{-06}$	$2.0E^{-07}$	$2.0E^{-09}$	$2.0E^{-09}$	$8.8E^{-06}$

Table S10 Prior and posterior parameter values obtained in DIYABC (Cornuet et al. 2008, 2010) in *Conyza canadensis* for Scenario 4 of scenario set C (Fig. 2C) using two different sets of five populations/multilocus genotypes, C2, b, a, d, and k and C2, b, a, h, and i as populations, P1 through P5, respectively. CI, credible interval; population size estimates (Nb2, Nb3, Nb4, Nb5, Nb, Nr, Nr3, Nr4, Nr5 and Ne) refer to the effective breeding population; µ, mean mutation rate; time in generations (t2, t3, t4, tr5, b3, b4, br5, t6, t1a, t1b, t7, t9, t10, t10h, and t11); *P*, mean coefficient of the geometric distribution under the Generalized Stepwise Mutation (GSM model); µsni, mean single nucleotide insertion-deletion mutation rate.

			Scenario 4 (C2,b,a,d,k)					Scenario 4 (C2,b,a,h,i)				
	prior values		mean	median	mode	95% CI		mean	median	mode	95% CI	
Resistant populations												
t2	1	150	109	116	145	38	149	101	106	137	29	148
t3	1	150	105	111	147	31	149	113	120	149	42	149
t4	1	150	111	118	149	41	149	115	123	145	44	149
tr5	1	40	32	34	40	14	40	31	33	40	12	40
b2	1	150	90	95	119	16	142	85	89	99	15	140
b3	1	150	87	92	109	12	141	93	99	116	17	142
b4	1	150	84	88	97	12	140	87	93	112	12	142
br5	1	40	22	23	25	3	38	22	22	26	3	38
r5	0.001	0.009	0.321	0.305	0.275	0.080	0.654	0.455	0.447	0.433	0.146	0.802
Nb2	2	100	13	7	2	2	67	16	9	2	2	73
Nb3	2	100	13	7	2	2	67	14	7	2	2	67
Nb4	2	100	15	8	2	2	72	17	9	3	2	78
Nb5	2	100	10	5	2	2	58	7	3	2	2	41
Nr	10	$1E^{06}$	46900	45600	4860	1740	96700	49800	50000	77800	1870	97500
Nr4	10	$1E^{06}$	49600	49200	28900	2140	97500	49400	49100	22400	2320	97400
Nr5	10	$1E^{06}$	53100	54500	94100	2910	97900	47300	45500	1950	2000	97300
Susceptible populations												
t1a	2	150	64	57	11	4	145	57	47	5	4	142
t1b	2	150	52	41	2	3	141	58	49	12	4	143
t7	150	10000	4350	4200	3830	461	8830	3990	3780	2450	444	8580
t9	150	10000	4180	3970	3360	592	8550	4310	4160	3230	553	8710
t6	150	10000	4450	4310	3900	801	8620	5390	5450	6250	1150	9150
r1	0.001	0.009	0.571	0.587	0.534	0.076	0.968	0.552	0.563	0.617	0.073	0.961
Nb	2	100	71	76	98	16	100	72	78	99	17	100

Resistant and susceptible populations												
ta	150	10000	8630	8950	9860	5690	9960	8570	8890	9810	5470	9960
Ne	10	10000	4050	3620	1530	403	9460	4190	3800	2350	451	9520
μ	$1E^{-05}$	$1E^{-04}$	$4.4E^{-05}$	$4.0E^{-05}$	$3.1E^{-05}$	$1.6E^{-05}$	$9.3E^{-05}$	$4.3E^{-05}$	$3.9E^{-05}$	$2.2E^{-05}$	$1.5E^{-05}$	$9.1E^{-05}$
P	$1E^{-01}$	$1E^{-07}$	$5.9E^{-01}$	$6.4E^{-01}$	$7.0E^{-01}$	$2.0E^{-01}$	$7.0E^{-01}$	$6.2E^{-01}$	$6.6E^{-01}$	$7.0E^{-01}$	$2.8E^{-01}$	$7.0E^{-01}$
μsni	$1E^{-09}$	$1E^{-05}$	$1.6E^{-06}$	$2.1E^{-07}$	$0.0E^{+00}$	$0.0E^{+00}$	$8.8E^{-06}$	$1.7E^{-06}$	$2.5E^{-07}$	$2.0E^{-09}$	$2.0E^{-09}$	$9.0E^{-06}$
